# Should rare and ultrarare diseases be treated differently in health coverage decisions?

**DOI:** 10.1186/s13023-026-04521-0

**Published:** 2026-08-05

**Authors:** Livia Fernandes Probst, Renata Cristina Ferreira Rola, Viviane Karoline da Silva Carvalho, Jorge Otávio Maia Barreto, Ivan Ricardo Zimmermann, Alfredo Palacios, Ávila Teixeira Vidal, Marisa Santos, Everton Nunes da Silva

**Affiliations:** 1 BP - A Beneficência Portuguesa de São Paulo, São Paulo, SP Brasil; 2 Programa de Pós-Graduação em Saúde Coletiva, Universidade de Brasília, Brasília, DF Brasil; 3 Fundação Oswaldo Cruz, Brasília, DF Brasil; 4 Faculdade de Ciências da Saúde, Universidade de Brasília, Brasília, DF Brasil; 5 Centre for Health Economics (CHE), University of York,York, England, UK; 6 Instituto Nacional de Cardiologia, Rio de Janeiro, RJ Brasil; 7 Faculdade de Ciências e Tecnologias em Saúde, Universidade de Brasília, Brasília, DF Brasil

**Keywords:** Health technology assessment, Rare diseases, Ultrarare diseases, Decision making, Health policy, Patient navigation, Health equity

## Abstract

**Background:**

Health technology assessment (HTA) is widely used to inform coverage and reimbursement decisions in health systems by integrating evidence on clinical effectiveness, safety, cost-effectiveness, and broader social considerations. However, the application of conventional HTA frameworks to rare diseases remains challenging due to limited clinical evidence, substantial uncertainty, and ongoing debates regarding the appropriateness of standard evaluation criteria. In this context, this study aimed to identify and synthesize additional criteria and their underlying justifications proposed and/or applied in HTA-based decision-making for rare diseases, and to examine the extent to which these criteria have been adopted by HTA agencies across countries.

**Methods:**

The protocol was registered in PROSPERO (CRD420251115351). We conducted an overview of reviews in accordance with the Cochrane Handbook and the PRIOR statement, complemented by a structured grey literature search of health technology assessment agencies and Ministries of Health affiliated with INAHTA and RedETSA. Systematic and scoping reviews, as well as institutional documents addressing criteria used in coverage decisions for rare diseases, were included. Findings were synthesized narratively, with criteria grouped into adapted standard and additional categories, and implementation patterns examined across different health system types.

**Results:**

Seventeen reviews and multiple institutional documents from eleven countries were included. Seven standard health technology assessment criteria and ten additional criteria were identified. Most jurisdictions reported adaptations of conventional health technology assessment approaches to address uncertainty and ethical concerns associated with rare diseases; however, only a limited number had operationalized a comprehensive set of additional criteria. Disease severity and therapeutic advantage were the most frequently applied additional criteria across countries, whereas treatment characteristics and risk-sharing mechanisms were inconsistently reported. Evidence specific to ultrarare diseases was scarcer, suggesting that decision-making for ultrarare conditions likely relies on criteria applied to rare diseases.

**Conclusions:**

Health technology assessment for rare and ultrarare diseases is characterized by selective and pragmatic adaptations rather than the use of unified alternative frameworks.

**Registration:**

PROSPERO CRD420251115351.

**Supplementary Information:**

The online version contains supplementary material available at https://doi.org/10.1186/s13023-026-04521-0.

## Highlights

This overview of reviews integrates published syntheses and grey literature to map how HTA criteria for rare and ultrarare diseases are proposed and operationalized across countries.

Most jurisdictions adapt standard HTA through selective modifiers, with disease severity and therapeutic advantage most frequently applied, while comprehensive rare-disease frameworks remain uncommon.

Findings highlight the need for greater transparency and governance on how additional criteria are incorporated, informing fairer, more consistent, and sustainable coverage decisions under uncertainty.

**Supplementary Information:**

The online version contains supplementary material available at https://doi.org/10.1186/s13023-026-04521-0.

## Background

Health technology assessment (HTA) is institutionalized in most health systems as a structured decision-support process to inform public coverage, reimbursement, and disinvestment decisions regarding health technologies [1]. HTA typically relies on a multidimensional appraisal framework that integrates evidence on health outcomes (efficacy, effectiveness, safety and quality of life), efficiency (cost-effectiveness), affordability (budget impact), and social and equity considerations [2].

Despite being the leading methodological reference for healthcare decision-making, conventional HTA frameworks presuppose levels of evidence generation and certainty that are often difficult to achieve in the context of rare diseases. These difficulties stem both from methodological features of HTA and from structural constraints affecting evidence production for rare conditions [3]. Clinical trials in rare and ultrarare diseases commonly involve small sample sizes, lack comparative arms, or rely on surrogate or non-validated outcomes, limiting the assessment of relative effectiveness and long-term benefits and resulting in substantial uncertainty regarding efficacy and safety [3, 4]. Consequently, HTA appraisals frequently face insufficient clinical evidence and limited understanding of disease natural history.

At the appraisal stage, additional methodological and normative challenges arise. These include concerns that the quality-adjusted life-year (QALY) may disadvantage individuals with irreversible disabilities due to limited potential health gains from low baseline utility; the lack of consensus on how equity considerations should be operationalized; and ongoing debate over whether willingness-to-pay thresholds should vary according to disease severity or unmet need [5–7]. Together, these challenges reflect the coexistence of empirical uncertainty and value-based tensions that complicate the application of standard HTA approaches to rare diseases [3].

In response, several authors and institutions have called for adaptations to HTA in the rare disease context, including greater flexibility regarding evidentiary requirements for safety, efficacy, and cost-effectiveness; the consideration of broader elements of value beyond health gain; formal inclusion of patient and caregiver perspectives in appraisal processes; and the use of higher or differentiated cost-effectiveness thresholds [8]. However, these proposals constitute a heterogeneous set of normative and procedural adaptations rather than a unified or consistently applied framework.

Such departures from standard HTA practice remain contested. Critics argue that adjusting evidence standards or valuing health gains differently solely based on rarity lacks a robust ethical, economic, or distributive justice justification [9, 10]. From this perspective, rarity alone should not confer preferential treatment, as it may undermine horizontal equity, obscure opportunity costs, and threaten the sustainability of healthcare systems; concerns central to broader debates on the special status of orphan drugs [11].

Against this background, no systematic synthesis has simultaneously consolidated the proposed adaptations to HTA for rare diseases and examined the extent to which they have been implemented by HTA agencies in practice. This overview of reviews aimed to identify and synthesize additional criteria and their underlying justifications proposed and/or applied in HTA-based decision-making for rare diseases, and to assess the extent to which these criteria have been adopted across countries. Additionally, the review examined the normative implications of these proposals, with particular attention to opportunity costs, potential reductions in overall population health, and alignment with established principles of efficiency and equity in priority setting.

## Methods

This overview was conducted in accordance with the Cochrane Handbook for Systematic Reviews of Interventions guidance for overviews of reviews [12]. Reporting followed the Preferred Reporting Items for Overviews of Reviews (PRIOR) statement (Supplementary material 1) [13]. The protocol was prospectively registered in PROSPERO (CRD420251115351).

Two complementary strategies were employed: (i) a scoping review of reviews and (ii) a structured grey literature search of institutional HTA documents. These approaches were designed to capture both conceptual discussions on criteria for rare diseases and their practical application in HTA decision-making.

### Eligibility criteria

The research question “*Should rare and ultrarare diseases be treated differently in terms of health technology coverage decisions?*” was framed using the PCC (Population, Concept, Context) framework (Table 1).

**Table 1:** Eligibility criteria based on the PCC framework

PCC element	Eligibility criteria
Population	Individuals living with rare or ultrarare diseases, irrespective of age, sex, or disease severity.
Concept	Any criterion explicitly proposed, discussed, or applied in decision-making processes related to the coverage of health technologies for rare or ultrarare diseases.
Context	National or regional health system–level decisions regarding the coverage, reimbursement, or disinvestment of health technologies.

Eligible evidence sources included: (i) systematic reviews, with or without meta-analyses; (ii) scoping reviews; and (iii) institutional documents produced by Ministries of Health or HTA agencies affiliated with INAHTA and/or RedETSA. Clinical decisions at the individual patient or hospital level were excluded, as they do not represent system-level coverage decisions.

### Information sources and search strategy

A comprehensive search of the published literature was conducted in MEDLINE (via PubMed), CINAHL, Embase, Scopus, Web of Science, the Cochrane Library, and Epistemonikos. An initial exploratory search in MEDLINE informed the development of a comprehensive strategy, which was subsequently adapted to each database according to its indexing system. Detailed strategies, executed on August 6, 2025, are presented in Supplementary material 2.

No language or country restrictions were applied. Studies published from 2000 onwards were eligible, reflecting the expansion of orphan drug policies and HTA institutionalization. This restriction was applied during study selection only.

Grey literature was identified through structured searches of publicly available documents from Ministries of Health and HTA agencies affiliated with INAHTA and/or RedETSA. A two-step approach was used: first, representatives of eligible agencies were contacted to complete an electronic questionnaire on regulatory frameworks and criteria for rare and ultrarare diseases; second, systematic searches were conducted on official institutional websites to complement and validate questionnaire responses.

### Selection process

All retrieved records were imported into Rayyan (Qatar Computing Research Institute, Doha, Qatar) for duplicate removal and screening [14]. Titles and abstracts, followed by full texts, were independently screened by two reviewers (LFP and RCFR). Disagreements were resolved by consensus.

Systematic reviews were required to report a clear research question, systematic methods, critical appraisal, and structured synthesis. Scoping reviews were included if they followed transparent search and selection procedures. No supplemental primary studies were included.

Grey literature selection was conducted by a single reviewer (VKSC) through manual screening of institutional websites. Documents were eligible if they were publicly available, produced or endorsed by governmental bodies or HTA agencies affiliated with INAHTA or RedETSA, and explicitly informed coverage or reimbursement decisions.

### Data collection process

Data from published literature were independently extracted by two reviewers (LFP and RCFR), while grey literature data were extracted by one reviewer (VKSC), using a standardized extraction form (Supplementary material 3). Disagreements were resolved by discussion or by consultation with a third reviewer (ENS). Corresponding authors were contacted when clarification or additional data were required.

### Data items

Extracted data included: (i) document and institutional characteristics; (ii) population focus and rarity definitions; (iii) detailed descriptions of criteria proposed or applied; (iv) underlying normative or justificatory rationales; and (v) evidence of formal adoption or operationalization in HTA or coverage decisions.

### Risk of bias assessment

Methodological quality and risk of bias of included reviews were assessed using the *JBI Critical Appraisal Checklist for Systematic Reviews and Research Synthesis* [15]. Two reviewers (LFP and RCFR) conducted assessments independently, with disagreements resolved by consensus.

### Reporting bias assessment

Information on the assessment of reporting bias was extracted from each included systematic review, including the use of funnel plots, statistical tests, or protocol comparisons. Findings were summarized descriptively and considered in the interpretation of results. As no new meta-analyses were conducted and no primary studies were included, no statistical assessment of reporting bias at the primary study level was performed.

### Synthesis methods

Findings were synthesized narratively due to heterogeneity in designs, objectives, and reporting, precluding quantitative meta-analysis. Evidence from systematic and scoping reviews was integrated with institutional documents to provide complementary perspectives.

Results were summarized according to definitions of rare and ultrarare diseases, standard HTA criteria, and additional or context-specific criteria. Tables and figures synthesized criteria, justifications, and evidence of adoption across countries. When available, findings specific to ultrarare diseases were analyzed separately.

Patterns were stratified by health system type (universal vs. non-universal) to highlight governance-related differences in HTA decision-making. Universal systems were defined as those guaranteeing population-wide entitlement financed predominantly through public or mandatory mechanisms, whereas non-universal systems relied on segmented or insurance-based arrangements [16, 17]. No statistical heterogeneity or sensitivity analyses were performed.

## Results

### Systematic review selection

**Fig. 1:**
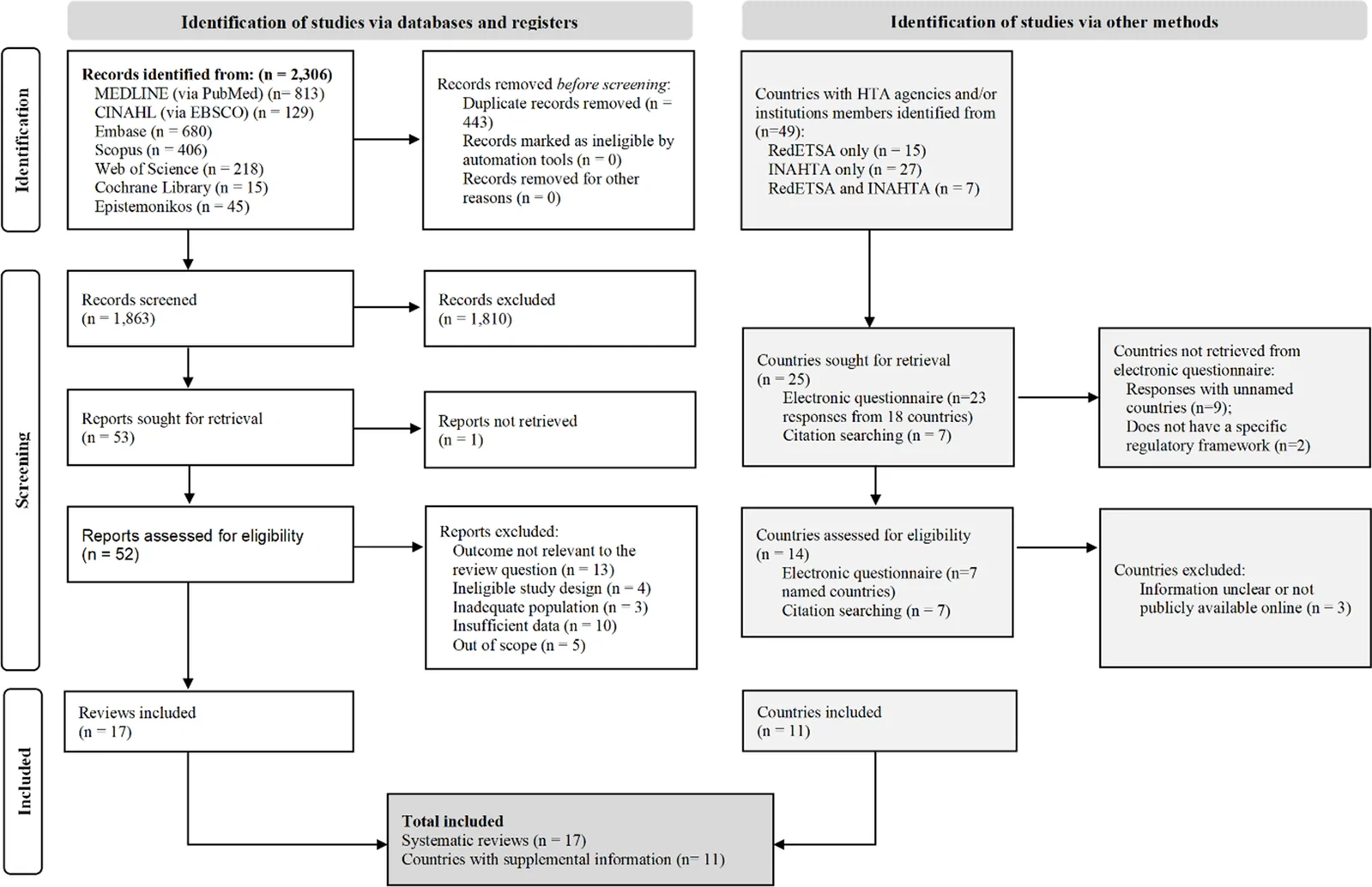
PRIOR FLOW diagram of the study selection process

Database searches identified 2,261 records. After duplicate removal, 1,858 references were screened by title and abstract, and 55 were assessed in full text. Seventeen reviews met the inclusion criteria and were included in this overview. The selection process is presented in Fig. 1 following the PRIOR flow diagram. A list of excluded full-text studies and institutional documents, with reasons for exclusion, is provided in Supplementary material 4.

### Characteristics of systematic reviews

The published evidence comprised ten systematic reviews [18–27] and seven scoping reviews [6, 28–33], offering complementary perspectives on methodological, ethical, and policy aspects of HTA for rare diseases. A detailed description of included studies is available in Supplementary material 5, with critical appraisal results and country classifications presented in Supplementary materials 6 and 7, respectively.

Grey literature included institutional documents from HTA agencies and Ministries of Health in 11 countries. Six had universal health systems (Australia, Brazil, Canada, New Zealand, and England and Scotland within the United Kingdom) [34–39], and five did not (France, Germany, Peru, Taiwan, and Belgium) [40–44]. These sources provided practical insights into how proposed criteria and value considerations are operationalized in HTA decision-making.

### Assessment criteria for rare diseases: a global perspective

Findings were organized into two overarching sets of criteria: (i) adapted standard HTA criteria and (ii) additional criteria proposed or applied specifically to rare or ultrarare diseases. The identified categories encompass different but complementary conceptual constructs, including assessment criteria, evidence considerations, and policy mechanisms that influence HTA decision-making. For the purposes of this review, these constructs were synthesized together because they were consistently reported in the literature as factors considered in the assessment of technologies for rare or ultrarare diseases.

The synthesis integrated two complementary sources of evidence. Institutional documents primarily described the intended policy frameworks, formal appraisal pathways, and decision-making criteria established by HTA agencies and Ministries of Health, whereas the published literature predominantly examined how these approaches were applied or evaluated. Accordingly, the findings reflects both the normative design of HTA systems and their reported implementation across jurisdictions.

Adapted standard criteria encompassed seven categories: clinical effectiveness and efficacy, evidence quality or certainty, safety profile, economic evaluation, cost-effectiveness thresholds, budget impact, and unmet health needs. Additional criteria included rarity, disease severity, stakeholder engagement, treatment features, therapeutic advantage, patient-reported outcome measures, humanistic or societal value considerations, alternative agreements, and the use of real-world evidence. Definitions for each criterion are presented in Table 2.

**Table 2:** Assessment criteria definition

HTA criteria	Definition and examples
**Adapted Standard Criteria in the Health Technology Assessment of Rare Disease Treatments**	
Clinical effectiveness and efficacy	Assesses clinically meaningful outcomes; in rare diseases, evidence is often limited, heterogeneous, and based on small samples or surrogate endpoints.
Evidence quality/ certainty	Evaluates the robustness and reliability of the evidence base, which for OMPs is frequently constrained by small, uncontrolled, or short-term studies, leading to substantial uncertainty.
Safety profile	Examines adverse events and the overall risk–benefit balance, often affected by limited long-term and comparative safety data.
Economic analysis	Applies ICERs, QALYs, and cost–utility analyses; in rare diseases, evidence is often weak, prompting flexible application or waiver of cost-effectiveness requirements.
Thresholds	Refers to willingness-to-pay or cost-effectiveness thresholds, which OMPs frequently exceed, leading to the use of higher, flexible, or implicit thresholds.
Budget impact	Assesses affordability and financial sustainability, often used as a proxy for opportunity cost in constrained health budgets.
Unmet health needs	Captures the absence of effective alternatives for severe or life-threatening conditions, commonly used to justify flexible evidence and reimbursement standards.
**Additional Criteria in the Health Technology Assessment of Rare Disease Treatments**	
Rarity	Considers low prevalence and very small target populations.
Disease severity	Reflects impact on mortality, morbidity, prognosis, and quality of life for patients and caregivers.
Stakeholders’ engagement	Incorporates input from patients, caregivers, clinicians, and other stakeholders into appraisal processes.
Treatment features	Accounts for treatment characteristics such as curative intent, life-long versus one-off use, and adherence-related aspects.
Therapeutic advantage	Assesses added therapeutic value compared with existing alternatives.
Patient-reported outcome measure	Considers patients’ self-reported symptoms, functioning, and quality of life.
Socioeconomic impacts	Captures broader social and economic consequences beyond health outcomes, such as effects on education, employment, and social participation.
Humanistic or societal value considerations	Reflects ethical and social values, including caregiver burden, productivity losses, supportive care needs, and equity considerations.
Risk sharing	Refers to financial or performance-based agreements (e.g., managed entry agreements, conditional reimbursement, expenditure caps) aimed at managing clinical and economic uncertainty.
Real-world evidence use	Involves post-launch data from routine clinical practice used to reduce evidentiary uncertainty and inform reassessment of value over time.

Legend: HTA, Health Technology Assessment; OMPs, orphan medicinal products; ICERs, incremental cost-effectiveness ratios; QALYs, quality-adjusted life years; WTP, willingness-to-pay

Across countries, clinical effectiveness remained the starting point for decision-making, even when evidence was limited, indirect, or based on surrogate outcomes [6, 22]. Evidence quality and uncertainty frequently triggered the use of managed entry agreements, post-marketing data collection, or real-world evidence requirements [20, 23, 30].

Cost-effectiveness criteria were often relaxed or waived due to difficulties in generating robust ICERs, with adaptive approaches observed in countries such as the United Kingdom, Canada, Italy, and South Korea [18, 26]. Budget impact consistently acted as a key moderating factor, reflecting affordability concerns [6, 20, 30]. Substantial unmet health needs also emerged as a central driver of flexibility, closely linked to equity principles and the rule of rescue [23, 28].

According to grey literature, specific HTA pathways for ultrarare diseases have only been identified in universal health systems, namely Australia, England, and Scotland [45]. Across countries, additional criteria were more frequently reported than adaptations of standard criteria, and no adaptations related to safety profile or treatment characteristics were identified. All countries described adaptations in economic evaluation and unmet health needs, including the acceptance of higher ICERs, greater evidence uncertainty, and increased weighting of QALYs [46]. Rarity, disease severity, and socioeconomic impact were the main additional criteria described to assess eligibility for the ultrarare disease pathway, as well as the broader impact of the technology beyond clinical benefits [47]. Overall, these findings highlight the use of criteria and decision-making modifiers that expand deliberations on equity, equality, uncertainty, and the prioritization of benefits despite high prices.

**Fig. 2:**
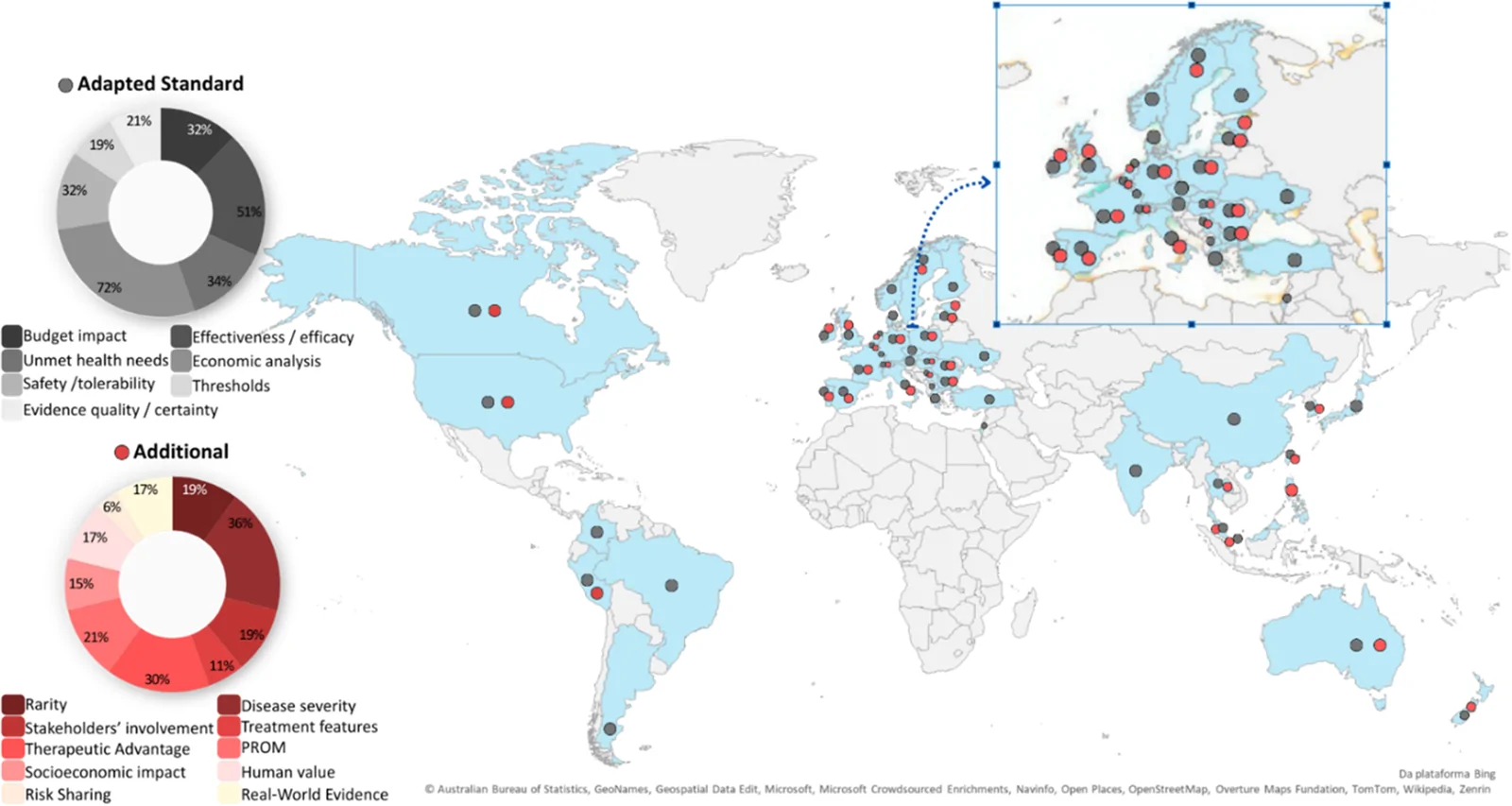
HTA criteria extension across countries

Figure 2 illustrates the geographic distribution of assessment criteria, showing a concentration of established HTA processes for rare diseases in Europe, with additional representation in North America, Oceania, Latin America, and Asia. Across regions, standard HTA frameworks remain foundational but are increasingly supplemented by additional criteria tailored to rare disease contexts.

### Justifications for adopting standard and additional criteria in the Health Technology Assessment of rare disease treatments

Countries justified the use of additional criteria by citing structural challenges in evidence generation, ethical imperatives, methodological limitations, and concerns about system sustainability. Evidence uncertainty, very small populations, lack of comparators, and high per-patient costs were frequently reported as reasons why standard HTA approaches were insufficient [6, 19, 20, 28, 33].

Ethical and social justifications included disease severity, lack of therapeutic alternatives, the rule of rescue, equity, solidarity, and prioritization of those “worst off” [22, 23, 27, 32]. Economic rationales emphasized fiscal sustainability, management of uncertainty, alignment of willingness-to-pay thresholds with national income levels, and responsible stewardship of public resources [18, 26, 30, 31].

Additional justifications related to innovation incentives, regulatory alignment, and support for transformative or first-in-class therapies [26, 27, 32]. Methodological rationales included challenges related to surrogate outcomes, validation of patient-reported measures, reliance on real-world evidence, and the use of conditional reimbursement or managed access schemes [29, 30].

Several jurisdictions also highlighted the importance of territorial equity, rapid access for severe conditions, administrative feasibility, transparency, and stakeholder inclusion [21, 22]. Overall, justifications converged on the need to balance equity, scientific rigor, sustainability, and social responsibility.

### Implementation status

Among the 47 countries identified through published and grey literature, 29 (62%) reported using additional criteria. Only Canada and the United Kingdom applied a broad and structured set of additional criteria, operationalizing 9 of the 10 categories identified. In most countries, additional criteria were implemented selectively, functioning as modifiers within standard appraisal pathways rather than as comprehensive frameworks. Disease severity (C2) and therapeutic advantage (C5) were the most frequently adopted criteria, while treatment features (C4) and risk-sharing mechanisms (C9) were less commonly reported. Table 3 summarizes implementation by country, with further details presented in Supplementary materials 8 and 9.

**Table 3:** Implementation status of additional criteria

Country	C1	C2	C3	C4	C5	C6	C7	C8	C9	C10	References
**Universal systems**											
Australia											[6, 20, 22, 30, 48–54]
Canada*											[6, 20, 21, 23, 28, 30, 48, 55–57]
Estonia											[20]
Ireland											[20, 30]
Latvia											[18, 20]
New Zealand											[20, 30, 38, 48, 58–61]
Portugal											[22]
Spain											[6, 20, 21, 23, 28, 30]
Sweden											[6, 23, 28]
UK**											[6, 18, 20, 22, 23, 28, 30, 31, 37, 46, 48, 62–72]
***Non-universal systems***											
Belgium											[20, 22, 23]
Bulgaria											[20, 23]
France											[48, 73–77]
Germany											[6, 19, 20, 22, 23, 30, 78–83]
Hungary											[20]
Italy											[6, 19, 20, 23]
Malaysia											[33]
Netherlands											[6, 20, 22, 23, 30]
Philippines											[24]
Poland											[18, 20, 23, 28]
Serbia											[20, 23]
Singapore											[24]
Slovakia											[20]
South Korea											[6]
Switzerland											[6, 22]
Peru											[44, 84]
Taiwan											[20, 43, 85–91]
Thailand											[24]
USA											[23]

Criteria: C1 – Rarity; C2 – Disease severity; C3 – Stakeholders’ engagement; C4 – Treatment features; C5 – Therapeutic advantage; C6 – Patient reported outcome measure; C7 – Socioeconomic impacts; C8 – Human value; C9 – Risk sharing; C10 – Real-World evidence utilization

Legend: blue – implemented; orange – implementation ongoing/ practical discussion; gray – theoretical discussion; white – Not implemented nor discussed

*It may be applicable only for a few provinces in Canada. ** It includes England, Scotland, Wales, and North Ireland. The criteria identified may not be applicable to all these countries

Countries with universal and non-universal health systems showed similar use of criteria related to disease severity, therapeutic advantage, stakeholder engagement, real-world evidence, and risk-sharing. Differences were more evident in the consideration of rarity, socioeconomic impact, and human value, which were more frequently reported in universal systems [38, 46–48, 53, 54, 56, 59, 63, 92–95]. In non-universal systems, only Peru, France, and Malaysia reported adaptations in this domain [44, 84, 96].

Therapeutic advantage was addressed by countries through innovative medicines or benefits demonstrated in analyses; however, explicit mentions of the use of a comparator were more frequent between countries without universal systems [43, 44, 74, 75, 79, 83, 84, 96–99]. The data collection after approval and financial agreements between payers and manufacturers have also been similarly reported, however, countries with universal systems tend to conduct negotiations on a case-by-case basis, outside the review process, or in a confidential manner [48, 54, 62].

Stakeholder engagement was widely implemented, either within standard HTA processes or through adapted mechanisms such as deliberative dialogues and consultations [44, 49–52, 55–57, 86, 96]. Engagement was also cited as a means to introduce flexibility into economic evaluations, particularly for technologies with high ICERs [49–52, 55–57, 83, 94, 100, 101].

Explicit guidance on patient-reported outcome measures was reported mainly by the United Kingdom, where NICE recommends supplementary quality-of-life evidence to assess the appropriateness of EQ-5D. In Germany, quality of life is considered alongside symptoms and adverse effects, provided validated instruments are used [79–81].

### Reporting biases

None of the included systematic reviews reported an assessment of reporting bias. No information was available on the use of funnel plots, statistical tests, or protocol comparisons. As a result, the potential influence of missing studies, analyses, or outcomes could not be determined. This limitation was documented and considered in the appraisal of the evidence and interpretation of findings.

## Discussion

### Main findings

This overview of reviews synthesized evidence from 17 systematic and scoping reviews and evidence from grey literature consisted of institutional documents produced by HTA agencies and Ministries of Health describing how countries adapt HTA and coverage decision making for rare diseases. The integration of institutional documents with published evidence allowed this review to distinguish between policy intent and policy implementation, providing a more comprehensive understanding of HTA approaches for rare diseases.

Across jurisdictions, rare disease policies and appraisal pathways were characterized by a tension between efficiency-oriented decision rules and the ethical and social imperatives typically invoked for conditions with high severity, limited treatment alternatives, and substantial uncertainty. In general, countries did not replace standard HTA with an entirely distinct model; instead, they introduced targeted adaptations, modifiers, or parallel routes.

A key finding of this review is that, although most countries report the use of additional criteria in the assessment of rare disease technologies, these criteria are implemented selectively rather than as part of a broad, structured framework. Disease severity and therapeutic advantage emerged as the most frequently applied additional criteria across countries, whereas treatment features and risk-sharing mechanisms were less consistently reported. This pattern suggests that, while some dimensions of additional value are routinely acknowledged, others remain sporadic or context dependent, reflecting limited formalization of rare disease–specific appraisal approaches.

An important conceptual consideration emerging from this review is that the identified categories do not represent a single type of construct. While some correspond to assessment criteria that directly inform value judgements, such as disease severity, unmet health needs, or therapeutic advantage, others relate to evidence considerations, including the acceptance of real-world evidence or lower levels of evidence, or to policy mechanisms, such as alternative reimbursement agreements. These constructs play distinct but complementary roles within HTA decision-making.

Comparative analysis between health system types showed that countries with universal and non-universal systems reported similar use of several additional criteria, including disease severity, therapeutic advantage, stakeholder engagement, real-world evidence use, and risk-sharing mechanisms. However, a key distinction lay in the consideration of rarity, socioeconomic impact, and human value. Countries with universal health systems more frequently reported incorporating broader social and socioeconomic considerations into HTA reviews, indicating a wider value perspective beyond clinical and economic domains. In contrast, among countries without universal health systems, only a small number, namely Peru, France, and Malaysia, reported adaptations in this domain, and these were generally less systematically described.

Overall, these findings indicate that rare disease decision making across jurisdictions remains largely driven by pragmatic deliberation and contextual judgement, rather than by standardized or comprehensive rare disease–specific frameworks. The selective adoption of additional criteria, combined with variation in how social and human value considerations are addressed, underscores ongoing challenges in achieving consistency, transparency, and equity in HTA processes for rare diseases [6, 20, 22, 31, 32].

### Interpretation in the context of other evidence

These findings are consistent with broader debates in the HTA literature about whether conventional cost-effectiveness approaches adequately capture value in rare diseases, and whether differential treatment can be justified without undermining equity, consistency, and opportunity cost principles [3, 4]. Prior normative analyses argue that rare diseases challenge HTA because evidence uncertainty is structural rather than incidental, and because social preferences may attach higher value to severity, unmet need, and fairness considerations that are incompletely represented in QALY based evaluations [102, 103]. At the same time, critics caution that creating exceptional routes or higher implicit thresholds may divert resources from interventions with larger population health gains, thereby generating new inequities [11].

Additionally, some countries justify the adoption of adapted or additional assessment criteria not only as a response to evidence limitations, but also as a strategy to address territorial equity and to stimulate innovation [21, 22, 26, 27, 32, 45, 59, 83, 104–106]. This justification can implicitly legitimize higher prices for rare-disease technologies as a necessary condition for commercial viability, thereby positioning governments as the main financier and risk-bearer for these products.

Public financing policies may foster innovation in technologies for rare diseases [107]. However, some studies demonstrate that the development of technologies for rare and ultrarare diseases has also become a lucrative niche for the pharmaceutical industry [108–111]. The European Union legislation for orphan drugs has been identified as a contributing factor in generating profitable returns for the pharmaceutical industries [108]. The regulation is grounded on the assumption that developing medicines for populations below 5 in 10 000 is not commercially profitable, while companies are not required to disclosure data on development costs [83, 108]. Regarding these aspects, specialists argue that the assessment of current financing models may be more effective in improving access to orphan drugs than developing new systems or criteria [111].

Our results reinforce that most countries are moving toward adaptive, deliberate and hybrid approaches rather than adopting a single alternative paradigm [112]. This partially aligns with the emergence of policy instruments such as managed entry agreements, coverage with evidence development, and formal consultation mechanisms as ways to reconcile uncertainty with timely access, while still maintaining some accountability to standard HTA principles [20, 102, 112].

### Limitations of the evidence and methods

Limitations must be acknowledged. First, the methodological quality of the included reviews was variable. Although most reviews reported clear objectives and adequate synthesis methods, recurrent limitations related to publication bias assessment and approaches to minimize errors in data extraction, and some reviews showed insufficient reporting across multiple items. Second, the evidence base is dominated by experiences from OECD and European settings, which may limit transferability to low- and middle-income contexts where institutional capacity, regulatory environments, and financing structures differ substantially [6, 20, 22].

Third, many included reviews relied primarily on publicly available policy documents and descriptive accounts of national decision-making processes. Consequently, the operational details regarding how criteria were weighted, how trade-offs were negotiated, and how final decisions were reached were often insufficiently documented, particularly when deliberations occurred outside formal appraisal reports or within confidential arrangements. In addition, information specific to ultrarare diseases was markedly scarce, which precluded a more in-depth analysis of these cases and limited the ability to assess whether decision-making for ultrarare conditions follows distinct criteria or largely mirrors those applied to rare diseases, despite the substantially greater clinical, evidentiary, and economic challenges involved.

An additional limitation relates to the comparative analysis between universal and non-universal health systems. Although this classification provided a pragmatic analytical framework for exploring broad international patterns in HTA approaches to rare diseases, it inevitably simplifies the substantial heterogeneity that exists within each group. Countries sharing the same health system model may differ considerably with respect to HTA maturity, governance, institutional arrangements, reimbursement policies, appraisal pathways, and decision-making processes. Therefore, the observed similarities and differences should be interpreted as overarching trends rather than uniform characteristics of all countries within each category. Future comparative studies adopting more granular classifications based on HTA governance or appraisal models may provide a more nuanced understanding of these contextual differences.

Methodological limitations of this overview should also be considered. Despite a comprehensive search strategy, relevant information may exist in non-indexed agency repositories, in grey literature not captured by the included sources, or in languages underrepresented in the selected reviews. In addition, the categorization of “additional criteria” necessarily involves interpretation, and boundaries between standard and additional criteria can be context dependent. The implementation status was inferred primarily from documented processes and may underrepresent informal practices or recent reforms not yet reflected in published sources. Furthermore, the identification, selection, and data extraction of institutional documents were conducted by a single reviewer. Although this approach is common in grey literature reviews, given the absence of standardized indexing and the exploratory nature of policy document identification, it may have increased the risk of selection bias and data extraction errors.

The reliance on secondary syntheses increases the risk of compounding biases already present in the source material. Additionally, some regions (e.g., Africa) remain underrepresented, limiting the generalizability of findings.

An additional limitation concerns the identification of structurally transformative measures, defined as reforms that meaningfully alter HTA appraisal processes. Although the search strategy captured formally documented frameworks, it may have failed to identify countries where adaptations are being tested through pilot initiatives, implementation experiments, or negotiated arrangements that are not consistently codified in HTA guidance or are reported only in fragmented grey literature. For example, in some settings, incremental changes appear to be advancing through implementation experiments, such as risk-sharing arrangements discussed in Brazil in the context of Zolgensma [113]. Consequently, our mapping may underestimate emerging, practice-based shifts and may not fully capture recent or pilot reforms that have not yet translated into stable, publicly accessible policy instruments.

### Implications for practice, policy, and research

The findings have implications for HTA agencies and policymakers seeking to strengthen legitimacy and consistency in rare disease decisions. First, the limited adoption of structured frameworks suggests a need to improve transparency on how additional criteria are defined, measured, and incorporated into deliberation. This includes clarifying decision rules for accepting uncertainty, specifying the role of severity and unmet need modifiers, and documenting how stakeholder input influences recommendations [6, 27, 29, 32].

Second, the uneven implementation of PROMs and real-world evidence points to a practical priority: strengthening data infrastructures and methodological guidance for outcomes meaningful to patients, including validated instruments and feasible real world collection strategies tailored to small populations [29]. For policy, the observed reliance on selective adaptations suggests that countries may benefit from formal governance arrangements that preserve consistency while allowing flexibility. This can include explicit criteria for eligibility to special routes, clearer standards for conditional reimbursement, and mechanisms to revisit decisions as new evidence emerges. Greater international dialogue may support convergence on minimal reporting standards for rare disease appraisals and on good practices for adaptive pathways, without imposing a single model across diverse health systems [6, 20, 32].

Future research should address three gaps. First, empirical studies are needed to examine how additional criteria are actually weighed in committee deliberations and how these tradeoffs affect funding decisions over time. Second, methodological work should test operational measures for broader value elements, including socio economic impacts and family burden, while minimizing double counting and preserving comparability. Third, comparative policy evaluations are needed to assess whether separate routes improve access and outcomes without exacerbating inequities or compromising sustainability. Ultimately, this overview indicates that progress has been made toward more context sensitive rare disease decisions, but substantial work remains to support fair, consistent, and sustainable coverage decisions under uncertainty. Strengthening data infrastructures, clarifying deliberative processes, and fostering international dialogue may support more consistent, legitimate, and accountable HTA practices for rare diseases under conditions of profound uncertainty.

## Conclusions

This overview demonstrates that, while health technology assessment frameworks for rare diseases remain anchored in conventional HTA principles, most countries have introduced selective adaptations to address the unique clinical, evidentiary, and ethical challenges posed by rare and ultrarare conditions.

Rather than adopting fully distinct appraisal models, jurisdictions tend to rely on pragmatic, hybrid approaches that combine standard criteria with context-dependent modifiers, negotiated arrangements, and adaptive evidence requirements. This heterogeneity underscores persistent tensions between efficiency, equity, and sustainability, and highlights the need for greater transparency, methodological clarity, and governance around the use of additional criteria in rare disease decision making.

### Registration and protocol

The protocol for this overview of reviews was prospectively registered in PROSPERO (CRD420251115351). The full protocol can be accessed through the PROSPERO registry. No amendments were made to the information provided at registration or to the protocol, and all procedures were conducted in accordance with the registered version.

## Supplementary Information

Below is the link to the electronic supplementary material.

Supplementary Material 1

## Data Availability

All data generated or analysed during this review will be included in the published article or be available by request from the study team.

## Abbreviations

HTA: Health Technology Assessment
ICERs: Incremental Cost-Effectiveness Ratios
OMPs: Orphan Medicinal Products
PCC: Population, Concept, Context
QALY: Quality-Adjusted Life-Year
WTP: Willingness-to-Pay
