# Supplementary material for: Should rare and ultrarare diseases be treated differently in health coverage decisions?
Source: Orphanet J Rare Dis. 2026 Aug 5;21:394. doi: 10.1186/s13023-026-04521-0 (PMC13732829; doi:10.1186/s13023-026-04521-0)
Supplement: Supplementary file 1 [file 13023_2026_4521_MOESM1_ESM.docx]

**Should rare and ultrarare diseases be treated differently in health coverage decisions? An overview of reviews**

**Supplemental Materials**

**Index of Additional file**

This document provides an index of the Supplementary Materials accompanying this research paper.

The Supplementary Materials include additional tables and data that could not be included in the main manuscript due to space constraints.

The materials are organized as follows:

[Supplementary material 1. Detailed search strategy by database (executed on 6 August 2025)	3](#_Toc224383232)

[Supplementary material 2. Predefined Tool Used for Data Extraction in the Scoping Review	4](#_Toc224383233)

[Supplementary material 3. Full-Text Articles and countries Excluded and Reasons for Exclusion	5](#_Toc224383234)

[Supplementary material 4. Characteristics of the Studies Included in the Review	8](#_Toc224383235)

[Supplementary material 5. Methodological quality of the included reviews	10](#_Toc224383236)

[Supplementary material 6. Included countries and classifications	12](#_Toc224383237)

[Supplementary material 7. Grey literature: Characteristics of HTA process for rare diseases by country	13](#_Toc224383238)

[Supplementary material 8. Grey literature: Adapted standard HTA criteria used by HTA agencies in coverage decisions for rare or ultra-rare diseases	17](#_Toc224383239)

[References of Supplementary material	21](#_Toc224383240)

Supplementary material 1. Detailed search strategy by database (executed on 6 August 2025)

| Database | Suggested strategy | # |
| --- | --- | --- |
| MEDLINE (via PubMed) | ("Rare Diseases"[Mesh] OR "Rare Disease*" OR "Orphan Disease*") AND ("Technology Assessment, Biomedical"[Mesh] OR "Biomedical Technology Assessment*" OR "Health Technology Assessment" OR "Technology Assessment" OR "Decision Making, Organizational"[Mesh] OR "Organizational Decision Making" OR "Decision Making"[Mesh] OR "Credit Assignment") | 813 |
| CINAHL (via EBSCO) | TI ("Rare Diseases" OR "Rare Disease*" OR "Orphan Disease*") AND TI ("Biomedical Technology Assessment*" OR "Health Technology Assessment*" OR "Technology Assessment*" OR "Organizational Decision Making" OR "Decision Making" OR "Credit Assignment") | 129 |
| Embase | ('rare disease'/exp OR 'orphan disease'/exp) AND ('biomedical technology assessment'/exp OR 'health assessment'/exp OR 'organizational decision making'/exp OR 'decision making'/exp) AND [embase]/lim NOT ([embase]/lim AND [medline]/lim) | 680 |
| Scopus | (TITLE-ABS-KEY("rare disease*" OR "orphan disease*") AND TITLE-ABS-KEY("health technology assessment" OR "biomedical technology assessment" OR "technology assessment" OR "organizational decision making" OR "decision making" OR "credit assignment")) AND ( LIMIT-TO ( DOCTYPE,"re" ) OR LIMIT-TO ( DOCTYPE,"cr" ) ) | 406 |
| Web of Science | "rare disease*" OR "orphan disease*"(All Fields) and "health technology assessment" OR "biomedical technology assessment" OR "technology assessment" OR "organizational decision making" OR "decision making" OR "credit assignment" (All Fields)  Refined By: Document Types: Review Article | 218 |
| Cochrane Library | #1 MeSH descriptor: [Rare Diseases] explode all trees  #2 #5 OR "orphan disease"  Refined By: only Cochrane reviews | 15 |
| Epistemonikos | (title:((title:("Rare Diseases" OR "Orphan Disease") OR abstract:("Rare Diseases" OR "Orphan Disease")) AND (title:("Biomedical Technology Assessment" OR "Health Technology Assessment" OR "Technology Assessment" OR "Organizational Decision Making" OR "Decision Making" OR "Credit Assignment") OR abstract:("Biomedical Technology Assessment" OR "Health Technology Assessment" OR "Technology Assessment" OR "Organizational Decision Making" OR "Decision Making" OR "Credit Assignment"))) OR abstract:((title:("Rare Diseases" OR "Orphan Disease") OR abstract:("Rare Diseases" OR "Orphan Disease")) AND (title:("Biomedical Technology Assessment" OR "Health Technology Assessment" OR "Technology Assessment" OR "Organizational Decision Making" OR "Decision Making" OR "Credit Assignment") OR abstract:("Biomedical Technology Assessment" OR "Health Technology Assessment" OR "Technology Assessment" OR "Organizational Decision Making" OR "Decision Making" OR "Credit Assignment")))) | 45 |
| TOTAL | | **2,306** |

Supplementary material 2. Predefined Tool Used for Data Extraction in the Scoping Review

| Category | Field | Description / Examples |
| --- | --- | --- |
| Identification | ***Document title*** | Full title of the source |
|  | ***Authors / Institution*** | Names of authors or responsible institutions (e.g., HTA agency) |
|  | ***Year of publication*** | Year when the document was published |
|  | ***Country / Region*** | Country or geographic context in which the document or framework was developed or applied |
|  | ***Type of source*** | Systematic review, HTA dossier, etc. |
|  | ***Source category*** | Academic literature, grey literature, agency website |
| Population | ***Rare disease(s) referenced*** | Specific rare disease(s) or group of diseases mentioned, if applicable |
|  | ***Disease prevalence*** | Stated prevalence or classification as "rare disease" (e.g., ≤ 5/10,000) |
|  | ***Age group*** | Paediatric, adult, mixed, or not specified |
| Concept | ***HTA criteria proposed or applied*** | Description of the specific criterion proposed or used for rare diseases (e.g., higher WTP threshold) |
|  | ***Rationale / Justification*** | Arguments provided to support differentiated treatment (e.g., ethical, equity, feasibility) |
|  | ***Source of the criterion*** | Stakeholder proposing or applying the change (e.g., agency, academic, patient group) |
|  | ***Broader value elements*** | Inclusion of criteria beyond standard cost-effectiveness (e.g., severity, burden of illness, innovation) |
|  | ***Method used to define criteria*** | Methodological approach (e.g., expert consensus, MCDA, Delphi, qualitative interviews) |
| Context | ***Decision level*** | Level at which the decision takes place: national, regional, hospital, supranational (e.g., European Union) |
|  | ***Health system type*** | Public, private, mixed; universal system vs. insurance-based |
|  | ***Jurisdiction / body*** | Institution responsible (e.g., NICE – UK, CADTH – Canada, CONITEC – Brazil, EMA – Europe) |
|  | ***Policy scope*** | Type of decision: adoption, coverage, reimbursement, disinvestment |
| Implementation | ***Evidence of adoption by HTA agencies*** | Whether the criterion has been implemented in real-world decision-making (Yes/No/Not reported) |
|  | ***Name of agency or program implementing*** | Name of the HTA body or institution implementing the criteria |
|  | ***Scope of application*** | Technology type (e.g., drug, gene therapy), level of decision (mandatory/optional) |
| Other | ***Stakeholder involvement*** | Participation of patients, caregivers, clinicians, etc., in the deliberative process |
|  | ***Limitations / critical remarks*** | Any concerns, limitations, or criticisms highlighted in the document regarding the proposed criteria |
|  | ***Additional comments / notes*** | Any other relevant information not captured above |

Supplementary material 3. Full-Text Articles and countries Excluded and Reasons for Exclusion

| Author (Year) | Title | Reason for Exclusion |
| --- | --- | --- |
| Abdallah et al., 2021 | Methodological Quality Assessment of Budget Impact Analyses for Orphan Drugs: A Systematic Review | Outcome not relevant: does not assess decision-making criteria or HTA processes. |
| Adkins et al., 2017 | Oncology drugs for orphan indications: how are HTA processes evolving for this specific drug category? | Outcome not relevant for the review question. |
| Aggarwal et al., 2022 | HTA83 Role of Quality of Life in Health Technology Assessment Agency Final Recommendations | Insufficient data: conference abstract only. |
| Ali et al., 2025 | Public preferences for allocating health system resources in Canada: a systematic review | Inadequate population: not restricted to rare diseases. |
| Angell et al., 2024 | Scoping Review of Economic Analyses of Rare Kidney Diseases | Outcome not relevant: does not examine decision-making processes. |
| Balijepalli et al., 2020 | Can Standard HTA Approaches Help Guide the Price of Orphan Drugs in Canada? | Ineligible study design: not a systematic or scoping review. |
| Baran-Kooiker et al., 2018 | MCDA Models in HTA of Orphan Drugs: A Systematic Literature Review | Outcome not relevant: methodological analysis not focused on decision-making for rare diseases. |
| Belancic et al., 2025 | A systematic review of economic evaluations of orphan medicines for SMA | Outcome not relevant for the review question. |
| Bloudek et al., 2021 | Are Drugs Priced in Accordance With Value? | Ineligible study design: not a systematic or scoping review. |
| Cannizzo et al., 2018 | Rare diseases under different levels of economic analysis | Outcome not relevant: does not examine decision-making criteria. |
| Chan et al., 2024 | The relative importance of severity and rarity criteria | Outcome not relevant for the review question. |
| Currie et al., 2023 | The Hidden Burden of Patients and Families in Rare Diseases | Insufficient data: conference abstract only. |
| Dangouloff et al., 2021 | Systematic literature review of economic burden of SMA | Outcome not relevant: does not assess decision-making frameworks. |
| Drummond et al., 2023 | How HTA bodies are responding to challenges of cell and gene therapy | Ineligible study design: narrative discussion. |
| Farris et al., 2023 | Economic evaluations for RPE65-mediated disease | Out of scope: does not address decision-making criteria. |
| Glaize et al., 2019 | Healthcare decision-making applications using MCDA | Inadequate population: results not specific to rare diseases. |
| Grand et al., 2024 | Issues, Challenges and Opportunities for Economic Evaluations of Orphan Drugs | Outcome not relevant: focuses on economic methods, not decision criteria. |
| Harricharan et al., 2023 | Special Considerations in HTA Processes for Rare Diseases | Insufficient data: conference abstract only. |
| Iskrov et al., 2017 | HTA and appraisal of therapies for rare diseases | Ineligible design: not systematic or scoping review. |
| Kanters et al., 2013 | Systematic review of high-priced inpatient orphan drugs | Outcome not relevant: no assessment of decision-making criteria. |
| Leonart et al., 2018 | Cost-effectiveness of acromegaly treatments | Outcome not relevant for the review question. |
| Meregaglia et al., 2023 | Estimation of utility values in rare diseases | Outcome not relevant: does not assess HTA decision-making. |
| Meregaglia et al., 2020 | Mapping health state utility values | Outcome not relevant: no evaluation of decision-making frameworks. |
| Mohammadshahi et al., 2022 | Methods and criteria for the assessment of orphan drugs | Outcome not relevant for the review question. |
| Morawski et al., 2014 | Reimbursement trends and evidence requirements for ultra-orphan therapies | Insufficient data: conference abstract only. |
| Palaska & Hutchings, 2015 | Value assessment and pricing frameworks for rare disease treatments | Insufficient data: conference abstract only. |
| Parada, 2024 | Systematic Literature Review of Funding Models | Insufficient data: conference abstract only. |
| Park, Griggs & Suh, 2015 | Cost Effectiveness of Monoclonal Antibody Therapy for Rare Diseases | Out of scope: does not address decision-making or HTA criteria. |
| Patikorn et al., 2025 | Role of Health Equity in HTA Processes: 13 Asian Health Systems | Inadequate population: not focused on rare diseases. |
| Perez et al., 2023 | Systematic Review for Rare Disease Policy in Peru and Colombia | Insufficient data: conference abstract only. |
| Quirland-Lazo et al., 2017 | Public policies and financing strategies for rare disease drugs | Insufficient data: conference abstract only. |
| Raycheva et al., 2023 | Challenges in mapping European rare disease databases | Out of scope: does not evaluate decision-making criteria. |
| Refolo et al., 2025 | Ethical, Legal and Social Assessment of AI-based Technologies for Rare Diseases | Out of scope: does not address HTA criteria for rare diseases. |
| Zelei et al., 2020 | Review of standard and non-standard value criteria for orphan drugs | Insufficient data: conference abstract only. |
| NICE HTA228 | Missed Opportunities in Cost-Effectiveness Analyses for Rare Disease Treatments | Insufficient data: conference abstract only. |
| Grey literature | | |
| Argentina | *Comisión Nacional de Evaluación de Tecnologías de Salud, Ministerio de Salud de la Nación* (CONETEC)  Ministry of Health  Legislation and/or government publications | Insufficient data: key information regarding the HTA process or criteria for rare or ultra-rare diseases has not been identified or the available information is unclear. |
| Mexico | Mexican government  Ministry of Health  *Centro Nacional de Excelencia Tecnológica en Salud* (CENETEC)  *Dirección General de Modernización del Sector Salud*(DGMoSS)  Legislation and/or government publications | Insufficient data: key information regarding the HTA process or criteria for rare or ultra-rare diseases has not been identified or the available information is unclear. |
| Uruguay | *Agencia de Evaluación de Tecnologías Sanitarias de Uruguay* (AETSU)  Ministry of Health  *Fondo Nacional de Recursos*  *Ministerio de Economía y Finanzas*  *Banco de Previsión Social (BPS)*  Legislation and/or government publications | Insufficient data: key information regarding the HTA process or criteria for rare or ultra-rare diseases has not been identified or the available information is unclear. |

Supplementary material 4. Characteristics of the Studies Included in the Review

| Study | Title | Country / Region | Type of publication |
| --- | --- | --- | --- |
| Czech et al 2020 [1] | A Review of Rare Disease Policies and Orphan Drug Reimbursement Systems in 12 Eurasian Countries | 12 Eurasian countries: Armenia, France, Germany, Kazakhstan, Latvia, Netherlands, Poland, Romania, Russia, Turkey, Ukraine, United Kingdom | Systematic review |
| Felippini et al 2024 [2] | HTA criteria adopted in different models of public healthcare systems for orphan drugs: A scoping review | Argentina, Australia, Brazil, Canada, Finland, France, Germany, Ireland, Italy, Malaysia, Netherlands, Poland, Russia, South Korea, Spain, Sweden, Switzerland, United Kingdom (England, Scotland, Wales, Northern Ireland) | Scoping review |
| Friedmann et al 2018 [3] | Using Multi-criteria Decision Analysis to Appraise Orphan Drugs: A Systematic Review | Bulgaria, Poland, United Kingdom, Spain, France, Russia, Netherlands, Germany, Italy, United States | Systematic review |
| Gammie et al 2015 [4] | Access to Orphan Drugs: A Comprehensive Review of Legislations, Regulations and Policies in 35 Countries | Australia; Austria; Belgium; Bulgaria; Canada; China; Czech Republic; Denmark; Estonia; Finland; France; Germany; Greece; Hungary; India; Ireland; Israel; Italy; Japan; Latvia; Macedonia; Poland; Portugal; Romania; Serbia; Singapore; Slovakia; Spain; Sweden; Switzerland; The Netherlands; Taiwan; Turkey; United Kingdom; United States. | Systematic review |
| Lasalvia et al 2019 [5] | International experiences in multicriteria decision analysis (MCDA) for evaluating orphan drugs: a scoping review | Canada, Poland, Spain, Belgium, Italy, United Kingdom, Netherlands, Norway, Bulgaria, Colombia | Scoping review |
| Menon et al 2015 [6] | Reimbursement of Drugs for Rare Diseases through the Public Healthcare System in Canada: Where Are We Now? | Canada | Systematic review |
| Nascimento et al 2023 [7] | Estratégias de Avaliação de Tecnologias em Saúde (ATS) para medicamentos para doenças raras: uma revisão rápida de escopo | Spain, EUA, EU, Ireland, Canada, Japan, Bulgaria, France, Germany, Italy, Netherlands, Poland, Russia, Ukraine, UK, Australia, Switzerland, Swiss, Scotland | Scoping review |
| Ng et al 2024 [8] | Comparative policy analysis of national rare disease funding policies in Australia, Singapore, South Korea, the United Kingdom and the United States: a scoping review. | Australia, Singapore, South Korea, United Kingdom, United States | Scoping review |
| Paulden et al 2014 [9] | Value-Based Reimbursement Decisions for Orphan Drugs: A Scoping Review and Decision Framework | Not country specific. The study is a scoping review synthesizing evidence and arguments from multiple countries, without extracting national results. Most examples and regulatory references come from OECD countries (Canada, EU, UK, Australia, US), but the findings are not organized by country. | Scoping review |
| Rahim et al 2024 [10] | Development of an multicriteria decision analysis framework for rare disease reimbursement prioritization in Malaysia | Malaysia | Scoping review |
| Short et al 20215 [11] | A National Approach to Reimbursement Decision-Making on Drugs for Rare Diseases in Canada? Insights from Across the Ponds | Canada (analysis based on 20 OECD countries: Australia, Austria, Belgium, Denmark, Finland, France, Germany, Iceland, Ireland, Italy, Japan, Korea, Luxembourg, Netherlands, New Zealand, Norway, Spain, Sweden, Switzerland, United Kingdom) | Systematic review |
| Vásquez et al 2024 [12] | Societal Preferences in Health Technology Assessments for Rare Diseases and Orphan Drugs: A Systematic Literature Review of New Analytic Approaches | Australia, Belgium, Bulgaria, Canada, Colombia, England, France, Germany, Hungary, Israel, Italy, Kazakhstan, The Netherlands, Norway, Poland, Romania, Russia, Spain, Sweden, Turkey, Ukraine, United Kingdom, United States | Systematic review |
| Whittal et al 2021 [13] | The Use of Patient‑Reported Outcome Measures in Rare Diseases and Implications for Health Technology Assessment | Not country-specific | Scoping review |
| Wong-Rieger et al 2025 [14] | Redefining value assessment and healthcare funding priorities for medicines: the journey to patient-centric decision making in APAC - a systematic literature review | Australia, China, Japan, Malaysia, New Zealand, Philippines, South Korea, Singapore, Taiwan, Thailand | Systematic review |
| Zelei et al 2021 [15] | Criteria and Scoring Functions Used in Multi-criteria Decision Analysis and Value Frameworks for the Assessment of Rare Disease Therapies: A Systematic Literature Review. | Not country-specific | Systematic review |
| Zelei et al 2016 [16] | Systematic review on the evaluation criteria of orphan medicines in Central and Eastern European countries | Bulgaria, Croatia, Czech Republic, Estonia, Hungary, Latvia, Lithuania, Poland, Romania, Slovakia, Slovenia, Serbia | Systematic review |
| Zimmermann et al 2021[17] | A systematic review of moral reasons on orphan drug reimbursement | Global systematic review, no specific country | Systematic review |

Supplementary material 5. Methodological quality of the included reviews

The methodological quality of the included reviews was appraised using the JBI Critical Appraisal Checklist for Systematic Reviews and Research Synthesis. Table 3 summarizes the assessments across the 17 reviews. Overall, most reviews adequately addressed key items such as clearly defined objectives, comprehensive search strategies, and appropriate methods for data synthesis (Q1–Q4, Q7–Q8, Q10–Q11). However, recurrent methodological limitations were observed in relation to the assessment of publication bias and the adequacy of strategies to minimize errors in data extraction (Q5–Q6), where several reviews were judged as “No”.” Some reviews[6,10] presented multiple items rated as “Unclear,” reflecting insufficient reporting. These findings indicate a variable methodological quality across the included reviews, with higher methodological robustness in more recent publications.[2,7,8]

**Risk of bias and methodological quality assessment of included reviews according to JBI Critical Appraisal Checklist for Systematic Reviews and Research Synthesis**

| **Study** | **Q1** | **Q2** | **Q3** | **Q4** | **Q5** | **Q6** | **Q7** | **Q8** | **Q9** | **Q10** | **Q11** |
| --- | --- | --- | --- | --- | --- | --- | --- | --- | --- | --- | --- |
| **Czech et al 2020[1]** | Y | Y | U | Y | N | N | Y | Y | N | Y | Y |
| **Felippini et al 2024[2]** | Y | Y | Y | Y | NA | NA | Y | Y | NA | Y | Y |
| **Friedmann et al 2018[3]** | Y | U | U | Y | N | N | U | Y | N | Y | Y |
| **Gammie et al 2015[4]** | Y | Y | Y | Y | N | N | N | Y | N | Y | Y |
| **Lasalvia et al 2019[5]** | Y | Y | Y | Y | NA | NA | Y | Y | NA | U | Y |
| **Menon et al 2015[6]** | U | N | Y | Y | N | N | Y | Y | N | Y | N |
| **Nascimento et al 2023[7]** | Y | Y | Y | Y | NA | NA | Y | Y | NA | Y | Y |
| **Ng et al 2024[8]** | Y | Y | Y | Y | NA | NA | U | Y | NA | Y | Y |
| **Paulden et al 2014[9]** | Y | Y | Y | Y | NA | NA | Y | Y | NA | Y | Y |
| **Rahim et al 2024[10]** | U | U | U | U | NA | NA | U | U | NA | Y | Y |
| **Short et al 2015[11]** | Y | Y | Y | Y | N | N | Y | Y | N | U | N |
| **Vásquez et al 2024[12]** | Y | Y | Y | Y | N | N | Y | Y | N | Y | Y |
| **Whittal et al 2021[13]** | Y | Y | U | U | NA | NA | Y | Y | NA | Y | Y |
| **Wong-Rieger et al2025 [14]** | Y | Y | Y | Y | N | N | Y | Y | N | Y | Y |
| **Zelei et al 2021[15]** | Y | Y | Y | Y | N | N | U | Y | N | Y | Y |
| **Zelei et al 2016[16]** | Y | U | Y | N | N | N | N | Y | N | Y | Y |
| **Zimmermann et al 2021[17]** | Y | U | Y | Y | N | N | U | Y | N | Y | Y |

**Notes:** Y: Yes; N: No; U: Unclear: NA: Not applicable. Q1. Is the review question clearly and explicitly stated?; Q2. Were the inclusion criteria appropriate for the review question?; Q3. Was the search strategy appropriate?; Q4. Were the sources and resources used to search for studies adequate?; Q5. Were the criteria for appraising studies appropriate?; Q6. Was critical appraisal conducted by two or more reviewers independently?; Q7. Were there methods to minimize errors in data extraction?; Q8. Were the methods used to combine studies appropriate?; Q9. Was the likelihood of publication bias assessed?; Q10. Were recommendations for policy and/or practice supported by the reported data?; Q11. Were the specific directives for new research appropriate?

Supplementary material 6. Included countries and classifications

The table below presents the classification of countries by healthcare system type, distinguishing between universal and non-universal systems. This classification was used to support comparative analyses of the use of additional criteria in health technology assessment processes and coverage decision-making for rare and ultrarare diseases across different institutional contexts.

| Country | Healthcare system classification |
| --- | --- |
| Australia | Universal system |
| Belgium | Non-universal system |
| Bulgaria | Non-universal system |
| Canada | Universal system |
| Estonia | Universal system |
| France | Non-universal system |
| Germany | Non-universal system |
| Hungary | Non-universal system |
| Ireland | Universal system |
| Italy | Non-universal system |
| Latvia | Universal system |
| Malaysia | Non-universal system |
| Netherlands | Non-universal system |
| New Zealand | Universal system |
| Peru | Non-universal system |
| Philippines | Non-universal system |
| Poland | Non-universal system |
| Portugal | Universal system |
| Serbia | Non-universal system |
| Singapore | Non-universal system |
| Slovakia | Non-universal system |
| South Korea | Non-universal system |
| Spain | Universal system |
| Sweden | Universal system |
| Switzerland | Non-universal system |
| Taiwan | Non-universal system |
| Thailand | Non-universal system |
| UK | Universal system |
| USA | Non-universal system |

Supplementary material 7. Grey literature: Characteristics of HTA process for rare diseases by country

Table below presents the main aspects of the processes used by HTA agencies for rare or ultra-rare diseases. Agencies can make the assessment of these technologies more flexible in two ways: by adapting their standard HTA process or by using a separate process for these diseases. In both situations, flexibility may be related to the analysis criteria or to changes in the evaluation process. The use of a special evaluation committee is an example of process alteration. Compared to the standard HTA process, the use of these committees may change the steps and the team responsible for the technology analysis or recommendation.[18–23] The committees may include representatives from government, patient and caregiver organizations, researchers and health experts, impartial citizens, and the pharmaceutical industry.[20–23] Across the countries observed, the committees are technical, have permanent members, and their use does not replace the stakeholder engagement stage.

The use of a separate assessment process for rare diseases was mentioned only by countries with no-universal health system. From this list, almost all have in progress processes related to, for example, implementation of assessments with other countries,[24–26] program review,[27,28] definition of methodological guidelines,[27–29] and legislative updates.[30–32] Separate processes for ultra-rare diseases, or ultra-orphan drugs, have only been found in countries with universal health systems. All of them have implemented processes, must be used in specific circumstances, and use the same prevalence threshold for ultra-rare disease (≤ 1/50,000 people),[33–39]

Adaptations to the standard HTA process were observed only in countries with a universal system. New Zealand was the only country that reported reviewing technologies for ultra-rare and rare diseases together, in an annual process conducted by the “*Pharmaceutical Management Agency*” (PHARMAC).[18,40] At present, their HTA processes are under development, as PHARMAC is aligning its definition of rare disease with the definition of the New Zealand Ministry of Health.[41,42]

The assessment timelines from the agencies indicate changes to the standard HTA process. For ultra-rare diseases or ultra-orphan drugs, the timelines are usually longer, and the entire process can take more than 3 years.[38,43,44] Australia follows a different path: the assessment can take up to 30 days,[39] a shorter period than the standard HTA average of 10 weeks.[45] In contrast to ultra-rare diseases, countries with separate processes for rare diseases may adopt shorter timelines than the standard HTA (4 to 34 weeks versus 25 to 42 weeks).[29,30,46–51] Across the countries that adapt their standard HTA process when they are dealing with rare or ultra-rare diseases, most didn’t report whether the assessment timelines are modified.

**Comparative overview of health technology assessment process for rare diseases/orphan drugs or ultra-rare diseases/drugs from grey literature**

| Country | Separate process - Rare diseases | Separate process - Ultra-Rare diseases | HTA adaptations - Rare diseases | HTA adaptations - Ultra-rare | Alternative / Special Access Program / Special authorization | Special Evaluation Committee | Assessment timelines (weeks)^1^ | Adoption by HTA agencies | References |
| --- | --- | --- | --- | --- | --- | --- | --- | --- | --- |
| *Countries with universal health systems* | | | | | | | | |  |
| Australia | No | Yes (LSDP) | Yes | NA | Not reported | Standard committee for rare diseases and the LSDP Expert panel for ultra-rare diseases | R: Not reported  U: 1 to 4  S: 10 (average time) | Implemented | [19,21,36,39,45,52] |
| Brazil | No | No | Yes | No | Not reported | Not reported | R: Not reported  S: 25 to 38 | Implemented | [53,54] |
| Canada | No | No | Yes | Not reported | SAP and province programs for rare diseases. | No, but the CDA may conduct the HTA of complex technologies with INESSS | R: Unclear, but it can be from 14 to 25 S: ≤ 25 | In progress | [52,55–63] |
| England (UK) | No | Yes (HST) | Yes | NA | Not reported | HSTEC for ultra-rare diseases | Unclear, but it can be (approximately)^2^:  U: 65 to 121  S: 13 to 61 | Implemented | [64–71] |
| New Zealand | No | No | Yes | Yes | NPPA | PTAC and Rare Disorders Advisory Committee | R: Not reported, but the submissions are annual  S: 93 (average time) | In progress | [18,40,42,72–77] |
| Scotland (UK) | No | Yes  (Ultra-orphan drugs) | Yes  (orphan drugs) | NA | Not reported | No (standard committees) | A: 22 to 26  U: can take > 3 years  S: ≤ 18 | Implemented | [37,38,44,52,78–81] |
| *Countries with no-universal health systems* | | | | | | | | |  |
| Belgium | Yes | No | NA | No | Special Solidarity Fund | Not reported | R: 12 to 25  S: ≤ 25 | In progress | [25,49,51,82–84] |
| Germany | Yes^3^ | No | NA | No | Not reported | No | R: Not reported  S: ≤ 13 | In progress | [26,52,85–88] |
| France | Yes, (AAP) | No | NA | No | Not reported | No | R: 12 to 25  S: ≤ 25 | In progress | [46,48,52,89–94] |
| Peru | Yes^4^ | No | NA | No | Temporary authorization | CFT and Institutional Advisory Commission (MoH) | R: 4 to 12  S: Not reported | In progress | [23,29,31,32,95,96] |
| Taiwan (Republic of China) | Yes (accelerated review) | No | NA | No | Ad-hoc authorization | Review Committee for Rare Diseases and Orphan Drugs | R: ≤ 34  S: 42 | Implemented | [22,47,50,97,98] |

Source: The authors

Legend: Not reported - Information about the subject wasn't found; Unclear - Some information was found, but the subject was unclear; In progress - Countries reported actions or adaptations that are being carried out; NA - Not applicable; Assessment Timelines^1^ (converted into weeks) - (R) Rare diseases or Orphan drugs, (U) Ultra-rare diseases or Ultra-orphan drugs, (S) Standard process.

**Note**: AAP - Early access authorisation (L’accès précoce); CDA - Canada’s Drug Agency; CFT - Pharmacotherapy Committees; HST - Highly Specialised Technologies process; HSTEC - Highly Specialised Technologies Evaluation Committee; HTA – Health Technology Assessment; INESSS - Institut national d’excellence en santé et en services sociaux (Québec); LSDP - Life Saving Drugs Program; MoH : Ministry of Health; NPPA - Named Patient Pharmaceutical Assessment; PTAC - Pharmacology and Therapeutics Advisory Committee; SAP - Special Access Program; UK - United Kingdom. England assessment timelines^2^: Estimation based on online documents retrieved by our general research. Separate Process - Germany^3^: for orphan drugs with annual revenue < €30 million; Peru^4^: for high-cost drugs for Rare/Orphan Diseases.

Supplementary material 8. Grey literature: Adapted standard HTA criteria used by HTA agencies in coverage decisions for rare or ultra-rare diseases

Table below presents the main standard criteria identified in grey literature. Overall, most countries reported flexibility regarding "unmet health needs," "economic analysis," and "evidence quality". All countries with universal systems reported adaptations to address "unmet health needs," such as adopting separate assessments, including additional analysis steps, or relaxing evidence requirements, especially when the drug is innovative or treatment alternatives are limited.[18–20,29,30,38,47,55,91,99]

Across countries with no universal system, adaptations regarding the “effectiveness /efficacy” of the technology were more frequent, including aspects related to potential drug effects and regulatory decisions.[31,91,94,100] In France, for example, the effectiveness and safety of a technology without marketing authorization can be presumed based on an opinion from the *French National Agency for Medicines and Health Products Safety* (ANSM).[92,101] Adaptations regarding safety were also directly reported. The *French National Authority for Health* (HAS) assumes that the early access authorization (AAP) may pose more risks to the patient than the traditional process, as it allows access to medicines without proven clinical benefit.[27,91] In Taiwan, special circumstances allow the manufacturer to submit a stability study for only one batch of the drug,[47] which may affect the safety, efficacy, and quality of the technology results.

**Adapted standard HTA criteria used by HTA agencies in coverage decisions for rare diseases/orphan drugs or ultra-rare diseases/drugs from grey literature**

| Country | C1 | C2 | C3 | C4 | | C5 | C6 | C7 | References |
| --- | --- | --- | --- | --- | --- | --- | --- | --- | --- |
| *Countries with universal health systems* | | | | |  | | | | |
| Australia | No | Yes | Yes | Yes | | No | No | No | [36,52] |
| Brazil | No  (Standard) | No  (Standard) | No  (Standard) | No  (Standard) | | No  (Standard) | Yes  (alternative thresholds up to three times higher than the standard) | No  (Standard) | [54] |
| Canada | No | No  (Standard) | Yes | No  (Standard) | | No  (Standard) | No, but some HTAs of drugs for rare diseases indicated a cost-effectiveness threshold of C$ 50,000/QALY. | Yes | [52,55,56] |
| England (UK) | Yes | No  (Standard) | Yes | Yes | | No | Yes (ICER < £100,000/QALY but can allow higher values)^1^ | Yes | [20,52,64,65,67,69,70] |
| New Zealand | No  (Standard) | No  (Standard) | Yes | No  (Standard) | | No  (Standard) | No | No | [18,52] |
| Scotland (UK) | No  (Standard) | No  (Standard) | Yes  (PACE meetings) | Yes | | No  (Standard) | No, but cite the UK threshold as a base for discussions. | Yes | [37,38,43,52,78,79,81,102] |
| *Countries with no-universal health systems* | | | | |  | | | | |
| Belgium | No | No  (Standard) | No | Yes | | No  (Standard) | No | No (Standard) | [25,51,84] |
| France | Unclear | Yes | Yes | Unclear | | Yes | No | No | [85,87,91,92,94,100,103–105] |
| Germany | No | Yes | No | No | | No | No  (only to define the HTA process) | Yes | [85,87,104] |
| Peru | No  (conducted by IAFAS) | Yes | Yes | Yes | | Yes | No  (only to define the HTA process) | Yes | [29,95,96] |
| Taiwan, Republic of China | No | Yes | Yes | Unclear | | Unclear | No | Yes | [47] |

Source: The authors.

**Notes:** C1 - Budget impact. C2 -Effectiveness / efficacy. C3 - Unmet health needs. C4 - Economic analysis. C5 - Safety / tolerability. C6 – Thresholds. C7 - Evidence quality / certainty.

**Legend**: Yes - any flexibilization or alteration from the standard was reported; No - no information or alteration from the standard process was reported or Information about the topic wasn't found; Unclear: Some information was found, but the topic was unclear; Blue – implemented; orange – implementation ongoing/ practical discussion; gray – theoretical discussion; white – Not implemented nor discussed.

Notes: IAFAS – Health Insurance Fund Administrators; PACE - Patient and Clinician Engagement; UK - United Kingdom. England thresholds^1^: For HST process.

Aspects regarding economic analysis and budget impact were reported similarly across countries with different health systems. In England, when the *National Institute for Health and Care Excellence* (NICE) are evaluating technologies for rare diseases, aspects such as treatment availability, rarity, disease severity, and level of evidence are considered before the cost-effectiveness.[33,106] Experts can be consulted to identify plausible values for modeling, and the committee can assign greater weight to QALY for severity, adopting the higher-level weighting (based on absolute and proportional deficit).[106] In Belgium, cost-effectiveness studies do not need to be included in HTA applications for orphan drugs.[51,84] Peru does not include a full economic evaluation for rare or orphan diseases,[96] but if the technology is high cost, the socioeconomic impact and cost-effectiveness should be assessed.[29,30,107]

Economic analysis for ultra-rare diseases can provide increased flexibility, particularly when contrasted with analyses conducted for rare diseases. In Australia, effective technologies that were previously rejected due to cost-effectiveness, may be reevaluated by the “*Life Saving Drugs Program* (LSDP)”, with flexible analysis of the patient needs and benefits of the technology.[19,36,39] Scotland, for example, could use modifiers during the assessment, assume a higher cost per QALY, and consider broader aspects, such as potential additional impacts of the technology at the individual and the health system levels.[37,52,78,79] England replaces standard HTA criteria, such as budget impact and quality of evidence, with HST eligibility criteria, accepting a higher level of uncertainty in the evidence.[20,71] Additionally, NICE may apply a different weight to QALYs, adopt a higher ICER, consider inequalities or factors unrelated to health, and use subjective judgment (e.g., "exceptionally negative impact").[20,71,106]

Cost-effectiveness and willingness to pay (WTP) thresholds have been formally established in England and in Brazil. NICE highlights that the higher the ICER, stronger the justification required for technology reimbursement must be, aiming for the effective use of resources.[20,106] The Brazilian National Commission for the Incorporation of Technologies in the National Health System (CONITEC) recently published guidelines about cost-effectiveness thresholds, recommending the use of an alternative threshold up to three times higher than the standard for rare diseases scenarios.[54]

In some cases, such as in Germany and Peru, thresholds were adopted only to define the assessment pathway that should be followed. In Germany, medicines with annual sales below 30 million euros do not need to attest clinical benefit.[26,85,87,108,109] In Peru, high-cost drugs for rare or orphan diseases (annual treatment cost per patient exceeding the limit of 8 tax units - UIT) must be submitted to a multi-criteria HTA.[29,30,107] Although it does not have formally defined thresholds, the *Scottish Medicines Consortium* (SMC) uses the NICE thresholds as a basis for discussions about an acceptable cost-benefit ratio.[37,79]

References of Supplementary material

1. Czech M, Baran-Kooiker A, Atikeler K, Demirtshyan M, Gaitova K, Holownia-Voloskova M, et al. A review of rare disease policies and orphan drug reimbursement systems in 12 Eurasian countries. Front Public Health. 2020;7:416. doi:10.3389/fpubh.2019.00416.
2. Felippini A, Biglia LV, Lima TM, Aguiar PM. HTA criteria adopted in different models of public healthcare systems for orphan drugs: a scoping review. Health Policy. 2024;144:105080. doi:10.1016/j.healthpol.2024.105080.
3. Friedmann C, Levy P, Hensel P, Hiligsmann M. Using multi-criteria decision analysis to appraise orphan drugs: a systematic review. Expert Rev Pharmacoecon Outcomes Res. 2018;18(2):135-146. doi:10.1080/14737167.2018.1414603.
4. Gammie T, Lu CY, Babar ZUD. Access to orphan drugs: a comprehensive review of legislations, regulations and policies in 35 countries. PLoS One. 2015;10:e0140002. doi:10.1371/journal.pone.0140002.
5. Lasalvia P, Prieto-Pinto L, Moreno M, Castrillón J, Romano G, Garzón-Orjuela N, et al. International experiences in multicriteria decision analysis (MCDA) for evaluating orphan drugs: a scoping review. Expert Rev Pharmacoecon Outcomes Res. 2019;19(4):409-420. doi:10.1080/14737167.2019.1633918.
6. Menon D, Clark D, Stafinski T. Reimbursement of drugs for rare diseases through the public healthcare system in Canada: where are we now? Healthc Policy. 2015;11(1):15-32. Available from: <http://www.ncbi.nlm.nih.gov/pubmed/26571466>
7. Nascimento LRS, Andrade MC, Zimmermann I. Estratégias de avaliação de tecnologias em saúde (ATS) para medicamentos para doenças raras: uma revisão rápida de escopo. J Bras Econ Saude. 2023;15(2):116-128. doi:10.21115/JBES.v15.n2.p116-28.
8. Ng QX, Ong C, Chan KE, Ong TSK, Lim IJX, Tang ASP, et al. Comparative policy analysis of national rare disease funding policies in Australia, Singapore, South Korea, the United Kingdom and the United States: a scoping review. Health Econ Rev. 2024;14:article number not informed. doi:10.1186/s13561-024-00519-1.
9. Paulden M, Stafinski T, Menon D, McCabe C. Value-based reimbursement decisions for orphan drugs: a scoping review and decision framework. Pharmacoeconomics. 2015;33(3):255-269. doi:10.1007/s40273-014-0235-x.
10. Ku Abd Rahim KN, Muhammad Lattepi N, Sarimin R, Foo SS, Akmal S, Lee SW, et al. Development of a multicriteria decision analysis framework for rare disease reimbursement prioritization in Malaysia. Int J Technol Assess Health Care. 2024;40:e31. doi:10.1017/S026646232400031X.
11. Short H, Stafinski T, Menon D. A national approach to reimbursement decision-making on drugs for rare diseases in Canada? Insights from across the ponds. Healthc Policy. 2015;10(4):24-46. Available from: <http://www.ncbi.nlm.nih.gov/pubmed/26142357>
12. Vásquez P, Hall L, Merlo G. Societal preferences in health technology assessments for rare diseases and orphan drugs: a systematic literature review of new analytic approaches. Value Health Reg Issues. 2024;44:101026. doi:10.1016/j.vhri.2024.101026.
13. Whittal A, Meregaglia M, Nicod E. The use of patient-reported outcome measures in rare diseases and implications for health technology assessment. Patient. 2021;14(4):485-503. doi:10.1007/s40271-020-00493-w.
14. Wong-Rieger D, Tsai IC, Tan JY, Wu DBC, Yu DY, Keetley A, et al. Redefining value assessment and healthcare funding priorities for medicines: the journey to patient-centric decision making in APAC - a systematic literature review. Int J Technol Assess Health Care. 2025;41:e22. doi:10.1017/S0266462325000224.
15. Zelei T, Mendola ND, Elezbawy B, Németh B, Campbell JD. Criteria and scoring functions used in multi-criteria decision analysis and value frameworks for the assessment of rare disease therapies: a systematic literature review. Pharmacoecon Open. 2021;5(4):605-612. doi:10.1007/s41669-021-00271-w.
16. Zelei T, Molnár MJ, Szegedi M, Kaló Z. Systematic review on the evaluation criteria of orphan medicines in Central and Eastern European countries. Orphanet J Rare Dis. 2016;11:72. doi:10.1186/s13023-016-0455-6.
17. Zimmermann BM, Eichinger J, Baumgartner MR. A systematic review of moral reasons on orphan drug reimbursement. Orphanet J Rare Dis. 2021;16:292. doi:10.1186/s13023-021-01925-y.
18. PHARMAC. Funding for rare disorders [Internet]. Wellington: Pharmaceutical Management Agency; 2025 [cited 2025 Dec 18]. Available from: <https://www.pharmac.govt.nz/medicine-funding-and-supply/the-funding-process/from-application-to-funded-medicine-how-we-fund-a-medicine/medicines-for-rare-disorders>
19. Australian Government Department of Health. Procedure guidance for medicines funded through the Life Saving Drugs Program (LSDP) [Internet]. Australia: Australian Government Department of Health, Disability and Ageing; 2018 [cited 2025 Dec 18]. Available from: <https://www.health.gov.au/resources/publications/procedure-guidance-for-medicines-funded-through-the-life-saving-drugs-program-lsdp?language=em>
20. NICE. Highly specialised technologies evaluation committee [Internet]. London: National Institute for Health and Care Excellence; 2025 [cited 2025 Oct 31]. Available from: <https://www.nice.org.uk/get-involved/our-committees/nice-committee-meetings/highly-specialised-technologies-evaluation-committee>
21. Department of Health Disability and Ageing. Life Saving Drugs Program Expert Panel [Internet]. Australia: Australian Government Department of Health, Disability and Ageing; 2025 [cited 2025 Dec 18]. Available from: <https://www.health.gov.au/committees-and-groups/life-saving-drugs-program-expert-panel>
22. Ministry of Health and Welfare. Prevention of Rare Diseases and Orphan Drug Act [Internet]. Taiwan: Ministry of Health and Welfare; 2015 Jan 14 [cited 2025 Dec 18]. Available from: <https://law.moj.gov.tw/ENG/LawClass/LawAll.aspx?pcode=L0030003>
23. Ministerio de Salud. Resolución Ministerial N.° 113-2022-MINSA: aprueba el Reglamento Interno de la Comisión Consultiva Institucional del Ministerio de Salud encargada de evaluar el diagnóstico y/o tratamiento de enfermedades raras y huérfanas (ERH) de alto costo [Internet]. Lima: Ministerio de Salud; 2022 [cited 2025 Dec 18]. Available from: <https://cdn.www.gob.pe/uploads/document/file/2855526/Resoluci%C3%B3n%20Ministerial%20%20N%C2%B0%20113-2022-MINSA%20.pdf?v=1645802938>
24. União Europeia. Regulamento (UE) 2021/2282 do Parlamento Europeu e do Conselho. J Off União Eur. 2021 Dec 15.
25. INAMI. Evaluation des médicaments (HTA) en collaboration avec d’autres pays européens [Internet]. Brussels: Institut national d’assurance maladie-invalidité; 2025 [cited 2025 Dec 18]. Available from: <https://www.inami.fgov.be/fr/themes/soins-de-sante-cout-et-remboursement/les-prestations-de-sante-que-vous-rembourse-votre-mutualite/medicaments/industrie-pharmaceutique/evaluation-des-medicaments-hta-en-collaboration-avec-d-autres-pays-europeens>
26. G-BA. The benefit assessment of medicinal products in accordance with the German Social Code, Book Five (SGB V), section 35a [Internet]. Berlin: Gemeinsamer Bundesausschuss; 2025 [cited 2025 Dec 18]. Available from: <https://www.g-ba.de/english/benefitassessment/>
27. HAS. Définition des conditions méthodologiques requises pour la prise d’un pari lors d’une demande d’accès précoce pré-AMM ou post-AMM conditionnelle, et sa levée: note de cadrage [Internet]. Paris: Haute Autorité de Santé; 2024 [cited 2025 Dec 19]. Available from: <https://www.has-sante.fr/jcms/p_3530711/en/definition-des-conditions-methodologiques-requises-pour-la-prise-d-un-pari-lors-d-une-demande-d-acces-precoce-pre-amm-ou-post-amm-conditionnelle-et-sa-levee-note-de-cadrage>
28. HAS. La HAS précise le cadre du « pari » de l’accès précoce aux médicaments [Internet]. Paris: Haute Autorité de Santé; 2024 [cited 2025 Dec 19]. Available from: <https://www.has-sante.fr/jcms/p_3525823/en/la-has-precise-le-cadre-du-pari-de-l-acces-precoce-aux-medicaments>
29. INS. Directiva Sanitaria No 001-INS/CETS-2025. Vs. 01. Metodología para la evaluación multicriterio para generar recomendaciones de uso de tecnologías sanitarias de alto costo para enfermedades raras o huérfanas [Internet]. Peru: Instituto Nacional de Salud, Centro de Evaluación de Tecnologías en Salud; 2025 [cited 2025 Dec 18]. p. 94. Available from: <https://www.gob.pe/institucion/ins/normas-legales/7249674-143-2025-ins-pe>
30. Government of Peru. Decreto Supremo N° 002-2025-SA que aprueba el Reglamento de la Ley N° 29698, Ley que declara de interés nacional y preferente atención el tratamiento de personas que padecen enfermedades raras o huérfanas. El Peruano. 2025 Mar 27.
31. Government of Peru. Decreto Supremo N° 017-2025-SA: incorpora la Segunda Disposición Complementaria Transitoria al Reglamento de la Ley N° 29698 [Internet]. 2025 [cited 2025 Dec 18]. Available from: <https://actualidadcivil.pe/norma/decreto-supremo-017-2025-sa/9a0c4cff-2eca-441c-bc90-fcddeeb6a1b4>
32. Government of Peru. Red Nacional de Evaluación de Tecnologías Sanitarias (RENETSA) [Internet]. Peru: Plataforma del Estado Peruano; 2025 [cited 2025 Dec 18]. Available from: <https://www.gob.pe/55329-red-nacional-de-evaluacion-de-tecnologias-sanitarias-renetsa>
33. NICE. NICE strategic principles: a complementary approach to public health, social care and rare disease topics. PMG46. London: National Institute for Health and Care Excellence; 2025 Apr.
34. PBS. Cost Recovery Fees and Charges [Internet]. Australia: Pharmaceutical Benefits Scheme, Australian Government Department of Health, Disability and Ageing; 2025 [cited 2025 Dec 19]. Available from: <https://www.pbs.gov.au/info/industry/listing/elements/fees-and-charges>
35. TGA. Meeting the eligibility criteria for orphan drug designation [Internet]. Australia: Therapeutic Goods Administration; 2024 [cited 2025 Dec 19]. Available from: <https://www.tga.gov.au/resources/guidance/meeting-eligibility-criteria-orphan-drug-designation>
36. Ministry of Health. Life Saving Drugs Program for medicine sponsors [Internet]. Australia: Australian Government Department of Health, Disability and Ageing; 2025 [cited 2025 Dec 18]. Available from: <https://www.health.gov.au/our-work/life-saving-drugs-program/for-medicine-sponsors#medicine-eligibility-criteria>
37. Scottish Medicines Consortium. Guidance to submitting companies for completion of new product assessment form (NPAF): supplement for medicines for extremely rare conditions (ultra-orphan medicines) [Internet]. Scotland: Scottish Medicines Consortium; 2025 Jul [cited 2025 Aug 31]. Available from: <https://scottishmedicines.org.uk/media/9351/2025-08-06-guidance-supplement-ultra-orphan-v30.docx>
38. Scottish Medicines Consortium. Patient and clinician engagement (PACE) meetings overview [Internet]. Scotland: Scottish Medicines Consortium; 2025 [cited 2025 Dec 18]. Available from: <https://scottishmedicines.org.uk/media/8998/pace-overview-v40.pdf>
39. Department of Health Disability and Ageing. About the Life Saving Drugs Program [Internet]. Australia: Australian Government Department of Health, Disability and Ageing; 2025 [cited 2025 Dec 18]. Available from: <https://www.health.gov.au/our-work/life-saving-drugs-program/about-the-lsdp>
40. PHARMAC. Rare disorders call for applications [Internet]. Wellington: PHARMAC; 2024 [cited 2025 Dec 18]. Available from: <https://www.pharmac.govt.nz/news-and-resources/consultations-and-decisions/2024-10-rare-disorders-call-for-applications>
41. PHARMAC. Proposal to change Pharmac’s policy principles for funding rare disorder medicines [Internet]. Wellington: PHARMAC; 2025 [cited 2025 Dec 20]. Available from: <https://www.pharmac.govt.nz/news-and-resources/consultations-and-decisions/2025-07-proposal-to-change-pharmacs-policy-principles-for-funding-rare-disorder-medicines>
42. Ministry of Health. Aotearoa New Zealand Rare Disorders Strategy - Te Rautaki o Aotearoa e Pā ana ki ngā Mate Mokorea [Internet]. Wellington: Ministry of Health; 2024 Jul [cited 2025 Dec 18]. Available from: <https://www.health.govt.nz/system/files/2024-07/hp8873_-_rare_disorders_v5.pdf>
43. Scottish Government. Ultra-orphan medicines pathway: guidance [Internet]. Edinburgh: Scottish Government; 2019 [cited 2025 Dec 18]. Available from: <https://www.gov.scot/publications/ultra-orphan-medicine-pathways-guidance/>
44. SMC. Timelines & submission scheduling [Internet]. Scotland: Scottish Medicines Consortium; 2025 [cited 2025 Dec 18]. Available from: <https://scottishmedicines.org.uk/making-a-submission/timelines-submission-scheduling/>
45. PBS. 6.2 Evaluation of submissions [Internet]. Australia: Pharmaceutical Benefits Scheme; 2025 [cited 2025 Dec 18]. Available from: <https://www.pbs.gov.au/info/industry/listing/procedure-guidance/6-consideration-submissions/6-2-evaluation-of-submissions>
46. HAS. Comprendre l’évaluation des médicaments [Internet]. Paris: Haute Autorité de Santé; 2021 [cited 2025 Dec 18]. Available from: <https://www.has-sante.fr/jcms/c_412115/fr/comprendre-l-evaluation-des-medicaments>
47. CDE. New Drug Application (NDA) [Internet]. Taiwan: Center for Drug Evaluation; 2025 [cited 2025 Oct 17]. Available from: <https://www.cde.org.tw/drugen/25797/26014/26084/26086/26092/normalPost>
48. HAS. Médicament en accès précoce: ce qu’il faut savoir [Internet]. Paris: Haute Autorité de Santé; 2023 [cited 2025 Dec 18]. Available from: <https://www.has-sante.fr/jcms/p_3274374/fr/medicament-en-acces-precoce-ce-qu-il-faut-savoir#toc_1_6>
49. RIZIV. Weesgeneesmiddelen [Internet]. Brussels: Rijksinstituut voor ziekte- en invaliditeitsverzekering; 2025 [cited 2025 Dec 18]. Available from: <https://www.riziv.fgov.be/nl/thema-s/verzorging-kosten-en-terugbetaling/wat-het-ziekenfonds-terugbetaalt/geneesmiddelen/geneesmiddel-terugbetalen/weesgeneesmiddelen>
50. TFDA. TFDA strengthens orphan drug access with policy reforms [Internet]. Taiwan: Taiwan Food and Drug Administration, Ministry of Health and Welfare; 2025 [cited 2025 Dec 18]. Available from: <https://www.fda.gov.tw/eng/newsContent.aspx?id=30970>
51. Sécurité sociale. Arrêté royal fixant les procédures, délais et conditions en matière d’intervention de l’assurance obligatoire soins de santé et indemnités dans le coût des spécialités pharmaceutiques [Internet]. Belgium: Sécurité sociale; 2018 Mar 15 [cited 2025 Dec 18]. Available from: <https://www.ejustice.just.fgov.be/cgi_loi/article.pl?language=fr&lg_txt=f&type=&sort=&numac_search=&cn_search=2018020122&caller=eli&&view_numac=2018020122nx2018020122fr>
52. CADTH. Drugs for rare diseases: a review of national and international health technology assessment agencies and public payers’ decision-making processes. CADTH Health Technology Review: Environmental Scan [Internet]. Ottawa: Canadian Agency for Drugs and Technologies in Health; 2021 [cited 2025 Aug 18]. p. 46. Available from: <https://www.cda-amc.ca/sites/default/files/es/es0355-drugs-for-rare-diseases-pw.pdf>
53. CONITEC. Perguntas frequentes [Internet]. Brasília: Comissão Nacional de Incorporação de Tecnologias no Sistema Único de Saúde; 2022 [cited 2026 Jan 29]. Available from: <https://www.gov.br/conitec/pt-br/acesso-a-informacao/perguntas-frequentes>
54. Ministério da Saúde. O uso de limiares de custo-efetividade nas decisões em saúde: recomendações da Comissão Nacional de Incorporação de Tecnologias no SUS [Internet]. Brasília: Ministério da Saúde, Secretaria de Ciência, Tecnologia, Inovação e Insumos Estratégicos em Saúde, Departamento de Gestão e Incorporação de Tecnologias em Saúde, Coordenação-Geral de Avaliação de Tecnologias em Saúde; 2022 [cited 2026 Jan 29]. Available from: <https://www.gov.br/conitec/pt-br/midias/pdf/2022/20221106_relatorio-uso-de-limiares-de-custo-efetividade-nas-decisoes-em-saude.pdf>
55. CDA-AMC. Procedures for reimbursement reviews. Ottawa: Canada’s Drug Agency; 2025.
56. CDA. Methods guide for health technology assessment [Internet]. Canada: Canada’s Drug Agency; 2025 [cited 2025 Dec 19]. Available from: <https://www.cda-amc.ca/sites/default/files/MG%20Methods/MG0030-Quantitative-Methods-Manual_Mar%202025.pdf>
57. CDA. Reimbursement recommendation: exagamglogene autotemcel (Casgevy). Can J Health Technol. 2025;5.
58. CDA. Fee schedule for pharmaceutical reviews [Internet]. Canada: Canada’s Drug Agency; 2025 Mar [cited 2025 Dec 20]. Available from: <https://www.cda-amc.ca/sites/default/files/Drug_Review_Process/Application_Fees_Pharmaceuticals.pdf>
59. CADTH. CADTH process for drugs with health system implications [Internet]. Canada: CADTH; 2020 [cited 2025 Dec 19]. Available from: <https://www.cda-amc.ca/sites/default/files/pdf/Drugs_Health_System_Implications.pdf>
60. CADTH. CADTH reimbursement recommendation: belzutifan (Welireg) [Internet]. Can J Health Technol. 2023 [cited 2025 Dec 20];3:24. Available from: <https://www.cda-amc.ca/sites/default/files/DRR/2023/PC0309%20Welireg%20-%20CADTH%20Final%20Recommendation%20Final.pdf>
61. CADTH. CADTH health technology review: an inventory of rare disease registries in the Canadian landscape [Internet]. Can J Health Technol. 2024 [cited 2025 Dec 20];4:71. Available from: <https://www.cda-amc.ca/sites/default/files/pdf/CP0033-Rare-Disease-Registries.pdf>
62. Health Canada. Health Canada’s special access programs: request a drug [Internet]. Ottawa: Health Canada; 2024 [cited 2025 Dec 20]. Available from: <https://www.canada.ca/en/health-canada/services/drugs-health-products/special-access/drugs.html>
63. Health Canada. The National Strategy for Drugs for Rare Diseases [Internet]. Ottawa: Health Canada; 2024 [cited 2025 Dec 20]. Available from: <https://www.canada.ca/en/health-canada/services/health-care-systems/national-pharmacare/strategy-drugs-rare-diseases.html>
64. NICE. Strategic principles for rare diseases. In: NICE strategic principles: a complementary approach to public health, social care and rare disease topics. PMG46 [Internet]. London: National Institute for Health and Care Excellence; 2025 [cited 2025 Oct 31]. p. 10. Available from: <https://www.nice.org.uk/process/pmg46/resources/nice-strategic-principles-a-complementary-approach-to-public-health-social-care-and-rare-disease-topics-13430355949/chapter/strategic-principles-for-rare-diseases>
65. NICE. Economic evaluation. In: NICE technology appraisal and highly specialised technologies guidance: the manual. PMG36 [Internet]. London: National Institute for Health and Care Excellence; 2025 [cited 2025 Dec 20]. Available from: <https://www.nice.org.uk/process/pmg36/chapter/economic-evaluation-2>
66. NICE. Developing the guidance. In: NICE technology appraisal and highly specialised technologies guidance: the manual [Internet]. London: National Institute for Health and Care Excellence; 2025 [cited 2025 Oct 31]. Available from: <https://www.nice.org.uk/process/pmg36/chapter/developing-the-guidance-2#patient-access-schemes-and-commercial-access-agreements-technology-appraisals-and-highly>
67. NICE. Committee recommendations. In: NICE technology appraisal and highly specialised technologies guidance: the manual. PMG36 [Internet]. London: National Institute for Health and Care Excellence; 2025 [cited 2025 Oct 31]. p. 194. Available from: <https://www.nice.org.uk/process/pmg36/resources/nice-technology-appraisal-and-highly-specialised-technologies-guidance-the-manual-pdf-72286779244741>
68. NICE. The vision. In: Highly specialised technologies: NICE prioritisation board routing criteria. In: NICE-wide topic prioritisation: the manual. PMG46 [Internet]. London: National Institute for Health and Care Excellence; 2025 [cited 2025 Oct 31]. p. 23. Available from: <https://www.nice.org.uk/process/pmg46/resources/highly-specialised-technologies-nice-prioritisation-board-routing-criteria-pdf-19830673472965>
69. NICE. HST routing criteria. In: Highly specialised technologies: NICE prioritisation board routing criteria. PMG46 [Internet]. London: National Institute for Health and Care Excellence; 2025 [cited 2025 Oct 31]. Available from: <https://www.nice.org.uk/process/pmg46/resources/highly-specialised-technologies-nice-prioritisation-board-routing-criteria-15301445581/chapter/hst-routing-criteria>
70. NICE. Routing deliberation and decision. In: Highly specialised technologies: NICE prioritisation board routing criteria. PMG46 [Internet]. London: National Institute for Health and Care Excellence; 2025 [cited 2025 Oct 31]. Available from: <https://www.nice.org.uk/process/pmg46/resources/highly-specialised-technologies-nice-prioritisation-board-routing-criteria-15301445581/chapter/routing-deliberation-and-decision>
71. NICE. Prioritisation framework stage 1 and stage 2 [Internet]. London: National Institute for Health and Care Excellence; 2025 [cited 2025 Oct 31]. Available from: <https://www.nice.org.uk/process/pmg46/chapter/prioritisation-framework-stage-1-and-stage-2>
72. PHARMAC. Exceptional Circumstances Framework [Internet]. Wellington: PHARMAC; 2020 [cited 2025 Dec 19]. Available from: <https://www.pharmac.govt.nz/medicine-funding-and-supply/the-funding-process/policies-manuals-and-processes/exceptional-circumstances-framework-including-the-named-patient-pharmaceutical-assessment-policy>
73. PHARMAC. Factors for consideration [Internet]. Wellington: PHARMAC; 2020 [cited 2025 Dec 19]. Available from: <https://www.pharmac.govt.nz/medicine-funding-and-supply/the-funding-process/policies-manuals-and-processes/factors-for-consideration>
74. PHARMAC. Pharmac review: progress update July 2025 [Internet]. Wellington: PHARMAC; 2025 [cited 2025 Dec 18]. Available from: <https://www.pharmac.govt.nz/about/reset-programme/pharmacreview/pharmac-review-progress-update-july-2025>
75. PHARMAC. NPPA applications [Internet]. Wellington: PHARMAC; 2024 [cited 2025 Dec 20]. Available from: <https://www.pharmac.govt.nz/medicine-funding-and-supply/make-an-application/nppa-applications>
76. PHARMAC. Pharmacology and Therapeutics Advisory Committee (PTAC) [Internet]. Wellington: PHARMAC; 2025 [cited 2025 Sep 16]. Available from: <https://www.pharmac.govt.nz/about/expert-advice/pharmacology-and-therapeutics-advisory-committee-ptac>
77. Ministry of Health. Health care and rare disorders: evidence brief [Internet]. New Zealand: Ministry of Health; 2024 Dec [cited 2025 Dec 20]. Available from: <https://www.health.govt.nz/system/files/2024-12/health-care-and-rare-disorders.pdf>
78. SMC. SMC modifiers used in appraising new medicines [Internet]. Scotland: Scottish Medicines Consortium; 2012 [cited 2025 Dec 18]. Available from: <https://scottishmedicines.org.uk/media/3565/modifiers.pdf>
79. SMC. A guide to quality adjusted life years (QALYs) [Internet]. Scotland: Scottish Medicines Consortium; 2018 [cited 2025 Dec 18]. p. 4. Available from: <https://scottishmedicines.org.uk/media/2839/guide-to-qalys.pdf>
80. SMC. A guide to the Scottish Medicines Consortium [Internet]. Scotland: Scottish Medicines Consortium; 2023 [cited 2025 Dec 19]. Available from: <https://scottishmedicines.org.uk/media/7605/20231818-a-guide-to-the-scottish-medicines-consortium.pdf>
81. Scottish Medicines Consortium. New product assessment form for ultra-orphan medicines [Internet]. Scotland: Scottish Medicines Consortium; 2025 [updated 2025 May; cited 2025 Aug 31]. Available from: <https://scottishmedicines.org.uk/media/9129/ultra-orphan-npaf-form.docx>
82. Sécurité sociale. Arrêté royal relatif au remboursement des médicaments orphelins et des spécialités pharmaceutiques remboursables dans le cadre d’une maladie rare [Internet]. Belgium: Service Public Fédéral Justice; 2018 Dec 19 [cited 2025 Dec 20]. Available from: <https://www.ejustice.just.fgov.be/cgi_loi/article.pl?language=fr&lg_txt=f&type=&sort=&numac_search=&cn_search=2018120612&caller=SUM&&view_numac=2018120612n>
83. INAMI. Le Fonds spécial de solidarité (FSS): remboursement exceptionnel de prestations médicales [Internet]. Brussels: Institut national d’assurance maladie-invalidité; 2025 [cited 2025 Dec 20]. Available from: <https://www.inami.fgov.be/fr/themes/soins-de-sante-cout-et-remboursement/facilites-financieres/fonds-special-de-solidarite>
84. Denis A, Simoens S, Fostier C, Mergaert L, Cleemput I. Policies for rare diseases and orphan drugs. Brussels: Belgian Health Care Knowledge Centre; 2009. (KCE report). doi:10.57598/R112C.
85. BFJ. § 35a Bewertung des Nutzens von Arzneimitteln mit neuen Wirkstoffen, Verordnungsermächtigung. In: Sozialgesetzbuch (SGB) Fünftes Buch (V) - Gesetzliche Krankenversicherung [Internet]. Germany: Bundesamt für Justiz; 2019 [cited 2025 Dec 19]. Available from: <https://www.gesetze-im-internet.de/sgb_5/__35a.html>
86. IQWiG. Dossier assessments [Internet]. Cologne: Institute for Quality and Efficiency in Health Care; 2025 [cited 2025 Dec 19]. Available from: <https://www.iqwig.de/en/about-us/methods/results/dossier-assessments/>
87. G-BA. Verfahrensordnung des Gemeinsamen Bundesausschusses: 5. Kapitel: Bewertung des Nutzens und der Kosten von Arzneimitteln nach §§ 35a und 35b SGB V. Berlin: Gemeinsamer Bundesausschuss; 2025. p. 153-218.
88. European Union. Regulation (EC) No 141/2000 of the European Parliament and of the Council of 16 December 1999 on orphan medicinal products [Internet]. Brussels: European Union; 2019 [cited 2025 Sep 4]. Available from: <https://eur-lex.europa.eu/eli/reg/2000/141/oj/eng>
89. HAS. Accès précoce des médicaments: accompagnement des laboratoires [Internet]. Paris: Haute Autorité de Santé; 2021 Sep [cited 2025 Dec 20]. Available from: <https://www.has-sante.fr/upload/docs/application/pdf/2021-06/acces_precoces_-_guide_accompagnement_des_laboratoires.pdf>
90. HAS. Comprendre l’évaluation économique des produits de santé [Internet]. Paris: Haute Autorité de Santé; 2023 [cited 2025 Dec 20]. Available from: <https://www.has-sante.fr/jcms/r_1502595/fr/comprendre-l-evaluation-economique-des-produits-de-sante>
91. HAS. Accès précoce à un médicament [Internet]. Paris: Haute Autorité de Santé; 2025 [cited 2025 Dec 18]. Available from: <https://www.has-sante.fr/jcms/r_1500918/fr/acces-precoce-a-un-medicament>
92. ANSM. Les accès précoces (AAP) [Internet]. Paris: Agence nationale de sécurité du médicament et des produits de santé; 2025 [cited 2025 Dec 18]. Available from: <https://ansm.sante.fr/documents/reference/medicaments-en-acces-derogatoires/les-acces-precoces-aap>
93. France. Code de la santé publique. Article R5121-68. Version en vigueur depuis le 1 juillet 2021 [Internet]. Paris: Légifrance; 2021 Jul 1 [cited 2025 Dec 20]. Available from: <https://www.legifrance.gouv.fr/codes/article_lc/LEGIARTI000026968481>
94. France. Code de la santé publique. Article L5121-12. In: Loi n° 2023-1250 du 26 décembre 2023 de financement de la sécurité sociale pour 2024 [Internet]. Paris: Légifrance; 2023 [cited 2025 Dec 18]. Available from: <https://www.legifrance.gouv.fr/loda/id/LEGIARTI000048670566/2023-12-28>
95. Government of Peru. Decreto Supremo N° 002-2025-SA: aprueba el Reglamento de la Ley N° 29698 [Internet]. El Peruano. 2025 Mar 27 [cited 2025 Dec 18]. Available from: <https://busquedas.elperuano.pe/dispositivo/NL/2384767-4>
96. Ministerio de Salud. Documento Técnico: Manual de Evaluación de Tecnologías Sanitarias - Versión Corta [Internet]. Peru: Ministerio de Salud; 2022 [cited 2025 Dec 18]. Available from: https://cdn.www.gob.pe/uploads/document/file/2857766/Documento%20T%C3%A9cnico%3A%20Manual%20de%20Evaluaci%C3%B3n%20de%20Tecnolog%C3%ADas%20Sanitarias-Versi%C3%B3n%20Corta.pdf?v=1645879248
97. NHIA. 新藥意見分享品項 [Internet]. Taiwan: National Health Insurance Administration, Ministry of Health and Welfare; 2019 [cited 2025 Dec 20]. p. 5. Available from: <https://www.nhi.gov.tw/ch/cp-7559-ee0b7-2466-1.html>
98. CDE. 加速健保藥品給付作業 [Internet]. Taiwan: Center for Drug Evaluation; 2024 [cited 2025 Dec 19]. Available from: <https://www.cde.org.tw/hta/1444/1832/1992/2001/18266/normalPost>
99. HAS. Accès précoces - infographie de la réforme: réforme de l’accès dérogatoire aux médicaments [Internet]. Paris: Haute Autorité de Santé; 2021 [cited 2025 Dec 19]. Available from: <https://www.has-sante.fr/upload/docs/application/pdf/2021-06/acces_precoces_-_infographie_de_la_reforme.pdf>
100. HAS. Qalsody: mise au point de la HAS [Internet]. Paris: Haute Autorité de Santé; 2024 [cited 2025 Dec 18]. Available from: <https://www.has-sante.fr/jcms/p_3555639/fr/qalsody-mise-au-point-de-la-has>
101. HAS. Early access to medicinal products [Internet]. Paris: Haute Autorité de Santé; 2021 [cited 2025 Dec 19]. Available from: <https://www.has-sante.fr/jcms/r_1500918/en/early-access-to-medicinal-products>
102. IMPACT-HTA. Country vignette of appraisal/reimbursement processes for rare disease medicines in Scotland. In: Nicod E, Whittal A, editors. IMPACT-HTA [Internet]. 2019 [cited 2025 Dec 19]. Available from: <https://8c3e11d9-5f36-452f-abe3-c95befd6e85d.filesusr.com/ugd/e1a359_74a19c766e544af090a2d43229cb6295.pdf>
103. HAS. Autorisation d’accès précoce aux médicaments: doctrine d’évaluation de la HAS. Guide version 2 [Internet]. Paris: Haute Autorité de Santé; 2022 [cited 2025 Sep 5]. p. 17. Available from: <https://www.has-sante.fr/upload/docs/application/pdf/2021-06/acces_precoces_-_doctrine.pdf>
104. IQWiG. Allgemeine Methoden - Entwurf für Version 8.0: 3 Nutzenbewertung medizinischer Interventionen [Internet]. Germany: Institute for Quality and Efficiency in Health Care; 2025 [cited 2025 Dec 19]. Available from: <https://www.iqwig.de/methoden/allgemeine-methoden_entwurf-fuer-version-8-0.pdf>
105. IQWiG. Allgemeine Methoden - Entwurf für Version 8.0: Anhang A rationale der Methodik zur Feststellung des Ausmaßes des Zusatznutzens [Internet]. Germany: Institute for Quality and Efficiency in Health Care; 2025 [cited 2025 Dec 19]. Available from: <https://www.iqwig.de/methoden/allgemeine-methoden_entwurf-fuer-version-8-0.pdf>
106. NICE. NICE technology appraisal and highly specialised technologies guidance: the manual. NICE process and methods. London: National Institute for Health and Care Excellence; 2025. p. 194.
107. Ministerio de Salud. Directiva Administrativa No 327-MINSA/DGIESP-2022. Establece los lineamientos para la determinación de las enfermedades raras o huérfanas de alto costo y la estimación del umbral de medicamentos de alto costo para las enfermedades raras o huérfanas. Peru: Ministerio de Salud; 2022.
108. G-BA. G-BA zum Tag der Seltenen Erkrankungen: „Wir nutzen unsere Kompetenzen für eine bessere medizinische Versorgung!“ [Internet]. Berlin: Gemeinsamer Bundesausschuss; 2021 [cited 2025 Dec 19]. Available from: <https://www.g-ba.de/presse/pressemitteilungen-meldungen/939/>
109. G-BA. Tragende Gründe zum Beschluss des Gemeinsamen Bundesausschusses über eine Änderung der Arzneimittel-Richtlinie: Anlage XII – Nutzenbewertung von Arzneimitteln mit neuen Wirkstoffen nach § 35a SGB V: Fosdenopterin. Berlin: Gemeinsamer Bundesausschuss; 2025.
